# Deep mutational scanning of CYP2C9, CYP2C19, and NUDT15 shows that pharmacogene variant interpretation requires assay-specific functional data

**DOI:** 10.1093/g3journal/jkag248

**Published:** 2026-08-29

**Authors:** Kevser Kübra Kırboğa

**Affiliations:** Faculty of Engineering, Department of Bioengineering, Bilecik Şeyh Edebali University, TR, Bilecik 11100, Türkiye

**Keywords:** pharmacogenomics, deep mutational scanning, function-specific variant effects, variant effect prediction, protein language models, AlphaMissense, CYP2C9, NUDT15

## Abstract

Pharmacogene missense variants can disrupt protein stability, catalytic competence, or substrate handling through distinct mechanisms. General-purpose predictors estimate clinical pathogenicity as a single scalar, whereas pharmacogene interpretation requires knowing which biochemical dimension a variant perturbs, since that determines whether reduced function is substrate-dependent. Five deep mutational scanning datasets comprising 26,198 missense variants across CYP2C9, CYP2C19, and NUDT15 were assembled from MaveDB. Paired assays showed that this dimensionality dominates the data: 28% of CYP2C9 variants (1,236 of 4,421) decoupled catalytic activity from abundance, and 48% of NUDT15 variants (1,364 of 2,844) decoupled thiopurine sensitivity from stability, with CYP2C9 discordance concentrating at substrate-channel residues. AlphaMissense, a representative general-purpose pathogenicity predictor, scored these classes in line with its clinical training objective rather than the assayed biochemistry, assigning likely-benign scores to 38 of 195 stable-but-dead CYP2C9 variants and likely-pathogenic scores to 140 of 222 destabilized but thiopurine-resistant NUDT15 variants. To test whether this dimensionality is recoverable, a supervised ESM-2 sequence baseline was benchmarked against the ESM1v zero-shot ensemble and AlphaMissense under position-based 5-fold cross-validation, together with 3 architectural extensions: AlphaFold structural features, multitask learning across paired assays, and contact-graph neural networks. The baseline reached Pearson *r* of 0.54–0.72, matching or marginally exceeding both comparators, and no extension improved upon it. Trained directly on each assay, it nonetheless recovered the paired-assay difference at *r* = 0.28 for CYP2C9 and 0.43 for NUDT15, separating discordant variants at AUROC 0.60 and 0.51. Pharmacogene interpretation therefore requires assay-specific, substrate-aware functional measurements rather than a single generic score.

## Graphical Abstract

**Graphical Abstract:**
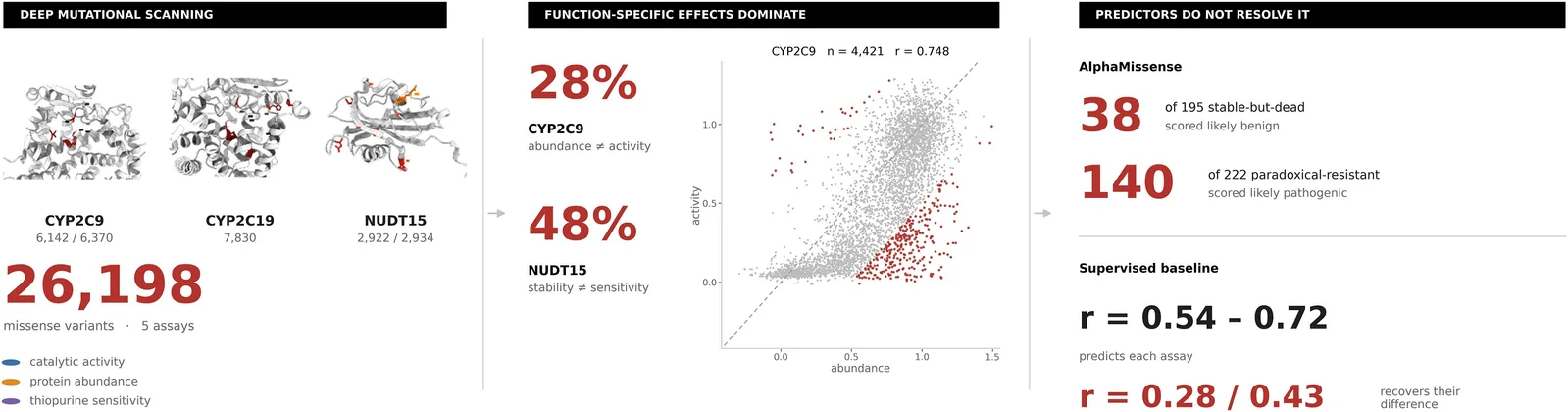
Function-specific effects dominate 26,198 pharmacogene missense variants across five deep mutational scanning datasets, and neither AlphaMissense nor a supervised sequence baseline resolves them.

## Introduction

Pharmacogenomics has entered clinical practice through established dosing guidelines that depend critically on the accurate functional classification of missense variants in drug-metabolizing enzymes, particularly members of the cytochrome P450 family (CYP2C9, CYP2C19, CYP2D6) and the thiopurine metabolism pathway (TPMT, NUDT15). Hundreds of new pharmacogene haplotypes are cataloged each year with unknown or uncertain functional consequences, and this growing gap between variant discovery and functional annotation has become a central bottleneck to the clinical implementation of pharmacogenomic testing. Brown and colleagues explicitly identified this bottleneck, emphasizing the need for high-throughput computational approaches that can keep pace with variant discovery in globally diverse populations (Brown et al. 2022), and although deep mutational scanning (DMS) data have since been generated for CYP2C9 (Amorosi et al. 2021), CYP2C19 (Boyle et al. 2024), and NUDT15 (Suiter et al. 2020), their integration into unified prediction frameworks has remained limited.

Existing computational approaches to variant interpretation fall into 3 broad categories, each contributing useful capabilities without fully resolving the pharmacogene-specific challenge. Zero-shot likelihood-based methods including ESM1v (Meier et al. 2021) and EVE (Frazer et al. 2021) leverage self-supervised protein language models to score variants without labeled data, while supervised models such as AlphaMissense (Cheng et al. 2023), Hubble.2D6 (Mcinnes et al. 2020), and VarDrug (Kariminejad et al. 2025) are trained directly on DMS or clinical phenotype labels, and optimized frameworks exemplified by Zhou et al. combine multiple algorithms through ensemble learning (Zhou et al. 2019). Despite this methodological diversity, all 3 categories share a fundamental constraint: they return a single scalar score per variant and cannot distinguish between the multiple biochemical dimensions along which a variant may act.

This single-score formulation is biologically inadequate for pharmacogenes, because a variant can perturb protein stability, catalytic competence, and substrate-specific binding through distinct molecular mechanisms that demand separate clinical interpretations. Boyle et al. documented that a subset of CYP2C9 variants preserves protein abundance while abolishing catalytic activity (Boyle et al. 2024), a pattern consistent with disruption of substrate binding rather than folding, and Maillard et al. reported additive effects of TPMT and NUDT15 variants on thiopurine toxicity in a multiethnic cohort of 1,768 patients (Maillard et al. 2024), demonstrating that substrate-specific sensitivity is clinically actionable independently of protein stability. Geier and colleagues generated 400 CYP2D6 double mutants at positions E216 and F483, measured hydroxylation activity against steroid substrates, and observed regioselectivity shifts that cannot be captured by any single functional score (Geier et al. 2012). Taken together, these observations point to a multidimensional structure of variant effect that existing predictors by construction cannot represent.

To address this gap, the present study first quantifies, directly from paired deep mutational scanning assays, the empirical magnitude of functional heterogeneity among missense variants in 3 clinically important pharmacogenes, and then asks whether that heterogeneity is recoverable by current computational predictors. Stratified analysis across biochemically defined variant categories establishes where the scores of a general-purpose pathogenicity predictor, AlphaMissense, diverge from assay-specific functional measurements. A reproducible supervised sequence baseline is then established using ESM-2 protein language model embeddings under position-based cross-validation and compared against the ESM1v zero-shot ensemble and AlphaMissense pretrained scores. Building on the resulting reference, 3 architectural extensions proposed as improvements over sequence-only baselines (structural feature augmentation from AlphaFold-predicted contact graphs, multitask joint learning across paired phenotypic assays, and graph neural network modeling of residue-residue contacts) are evaluated under identical splits and paired significance testing. The benchmark that emerges serves primarily as evidence for the scope of the interpretive problem, characterizing which variant categories remain inaccessible to current predictors without additional biochemical context. A complete framework integrating small-molecule substrate representations and nonadditive haplotype-level interactions is reserved for future work, when the requisite multisubstrate DMS and double-mutant datasets become available.

## Materials and methods

### Data acquisition

Five deep mutational scanning datasets were retrieved from the MaveDB repository (https://api.mavedb.org/api/v1) in January 2026. For CYP2C9 (UniProt P11712), we obtained activity scores measured by Click-seq ((Amorosi et al. 2021); urn:mavedb:00000095-a-1; *n* = 6,524 entries) and abundance scores measured by VAMP-seq (urn:mavedb:00000095-b-1; *n* = 6,821 entries). For CYP2C19 (UniProt P33261), we obtained VAMP-seq abundance scores ((Boyle et al. 2024); urn:mavedb:00001199-a-1; *n* = 8,149 entries, with 3 technical replicates). For NUDT15 (UniProt Q9NV35), we obtained protein stability scores (urn:mavedb:00000055-a-1; *n* = 3,100), thiopurine sensitivity scores (urn:mavedb:00000055-b-1; *n* = 3,100), and a combined dataset containing both measurements per variant (urn:mavedb:00000055-0-1; *n* = 3,015 entries with paired abundance_score and sensitivity_score columns) from Suiter et al. (2020). Canonical wild-type protein sequences were retrieved from UniProt via REST API. A search of MaveDB for these 3 genes returned 3 further score sets that were not used: 2 abundance datasets of 109 CYP2C9 and 121 CYP2C19 variants (urn:mavedb:00000062-a-1 and urn:mavedb:00000062-b-1), which are more than an order of magnitude smaller than the saturation datasets analyzed here and cover the same biochemical property, and a set of CYP2C19 synonymous scores (urn:mavedb:00001199-a-2), which contains no missense variants. The 5 datasets listed in Table 1 are therefore the complete set of saturation-scale missense score sets available for these genes. The same search returned no score set for CYP2D6. An overview of the datasets and their coverage statistics is provided in Table 1.

The NUDT15 thiopurine assay is referred to throughout as sensitivity rather than activity, since it measures the cellular thiopurine phenotype conferred by a variant rather than nucleotide hydrolase activity directly, and the 2 are not equivalent measurements. As with the other 4 datasets the score is normalized so that wild-type is approximately 1 and nonsense approximately 0; because wild-type NUDT15 degrades thiopurine nucleotides and thereby confers tolerance, a high score corresponds to preserved thiopurine tolerance and a low score to thiopurine sensitivity.

### Variant parsing and wild-type validation

HGVS protein nomenclature strings were parsed using a regular expression pattern matching single missense variants (e.g. p. Ala123Val), with synonymous (p. Ala123=) and nonsense (p. Ala123Ter) variants excluded from supervised modeling. Three-letter amino acid codes were mapped to single-letter representations using the standard IUPAC dictionary. To ensure numbering consistency between DMS data and UniProt reference sequences, we cross-validated wild-type amino acid identity at every unique position present in each DMS dataset. All 3 proteins achieved 100% wild-type concordance (CYP2C9: 477 of 477 positions; CYP2C19: 486 of 486 positions; NUDT15: 163 of 163 positions), confirming that no isoform or numbering correction was required.

### Exploratory analysis of functional heterogeneity

For CYP2C9, we joined the activity and abundance datasets on HGVS notation, yielding 4,421 variants with paired measurements across both assays. The Pearson correlation coefficient was computed on the full joint distribution, and variants were classified as function-specific when the absolute difference between activity and abundance scores exceeded 0.3 normalized units. For NUDT15, abundance_score and sensitivity_score columns from the combined dataset were directly compared across the 2,844 variants with nonmissing values in both columns. Because both the function-specific classification and the biochemical category definitions used in Section “A general-purpose pathogenicity predictor does not separate biochemically distinct variant categories” depend on threshold choices, the robustness of these definitions was assessed by repeating the classification across a range of cutoffs. The absolute-difference criterion was varied from 0.10 to 0.60 in steps of 0.05 for both proteins, and the joint category definitions were varied across 5 paired cutoff combinations per protein, spanning abundance cutoffs from 0.60 to 0.80 against activity cutoffs from 0.40 to 0.20 for CYP2C9, and sensitivity cutoffs from 0.70 to 0.90 against abundance cutoffs from 0.40 to 0.20 for NUDT15. Results of these sweeps are reported in Supplementary Tables 1 and 2, described in Sections “Functional heterogeneity in CYP2C9 and NUDT15 and A general-purpose pathogenicity predictor does not separate biochemically distinct variant categories”. The Pearson correlations between paired assays, which constitute the primary quantitative evidence for decoupling, involve no threshold and are unaffected by these choices.

### Protein language model embeddings

Wild-type per-residue embeddings were extracted using ESM-2 (esm2_t33_650M_UR50D;(Lin et al. 2023)) implemented in the fair-esm package (version 2.0.0). Each protein sequence was encoded as a single batch with beginning-of-sequence and end-of-sequence tokens, passed through the model in evaluation mode on an NVIDIA RTX 4060 Laptop GPU (8.59 GB VRAM), and the output representation at layer 33 (dimension 1280) was extracted for each residue after removal of the special tokens. Embeddings were cached to disk as PyTorch tensor files for reuse across downstream experiments. Peak GPU memory utilization during embedding extraction did not exceed 2.74 GB for the longest sequence tested (490 residues).

### Feature representations

Five feature variants were constructed to evaluate the relative contribution of sequence context, amino acid identity, spatial neighborhood, and global sequence statistics. The base variant (F1) used only the ESM-2 per-residue embedding at the variant position (dimension 1280). Variant F2 concatenated F1 with one-hot encodings of the wild-type and mutant amino acid identities (dimension 1320). Variant F3 replaced the single position embedding with a ±2-residue window of concatenated embeddings, combined with one-hot encodings (dimension 6440, with zero-padding at sequence boundaries). Variant F4 augmented F2 with both the per-position embedding offset from the mean and the mean itself, yielding a mean-referenced representation (dimension 3880). Variant F5 combined all preceding components (dimension 9000). All 5 feature variants are built from wild-type per-residue embeddings rather than from embeddings of the mutant sequence, for 2 reasons. Computationally, a wild-type embedding requires a single forward pass per protein and can be cached and reused across every variant at that protein, whereas mutant-sequence embeddings or delta embeddings would require one forward pass per unique mutant sequence, raising the extraction cost from 3 forward passes in total to several thousand per protein on the single 8 GB GPU used here (Section “Protein language model embeddings”). As a matter of design, the wild-type embedding at the variant position concatenated with wild-type and mutant amino acid encodings constitutes a minimal representation whose components can be ablated cleanly, which is the purpose of the F1 to F5 comparison. Mutant-sequence embeddings, delta embeddings, likelihood-ratio features derived from protein language models, and lightweight fine-tuning strategies such as adapter or low-rank updates are plausible alternatives that were not evaluated here, and their omission is discussed among the limitations of this study.

### Model architecture

All variants were evaluated with a two-layer multilayer perceptron (MLP) with ReLU activations and dropout regularization (*P* = 0.2). Hidden layer widths were adjusted to input dimensionality: inputs of dimension ≤ 2,000 used hidden layers of 256 and 64 units, while higher-dimensional inputs used 512 and 128 units. The output layer produced a single scalar prediction. Models were optimized using Adam (learning rate 1 × 10^−3^, weight decay 1 × 10^−5^) with mean squared error loss, batch size 128, and early stopping on validation Pearson correlation with a patience of 12 epochs. Because hidden-layer width was tied to input dimensionality in this way, feature variants differ from one another in both the information they encode and the capacity of the network that consumes it, and the 2 were not varied independently. Differences between feature variants should therefore be read as differences between complete configurations rather than as differences attributable to feature content alone.

### Cross-validation protocol

To prevent information leakage between training and evaluation sets, we implemented position-based cross-validation, in which all variants occurring at the same residue position were assigned to the same fold. Five-fold cross-validation was performed using scikit-learn's KFold with shuffle = True and random_state = 42, splitting at the position level rather than the variant level. Within each training fold, 20% of positions were further held out as a validation set for early stopping, using per-fold seeds to ensure determinism across reruns. For full reproducibility, cuDNN deterministic mode was enabled, and CUBLAS workspace was configured (CUBLAS_WORKSPACE_CONFIG=":4096:8").

### Evaluation metrics

For each held-out test fold, we computed 4 metrics: mean squared error (MSE), Pearson correlation coefficient (*r*), Spearman rank correlation (*ρ*), and coefficient of determination (*R*^2^ = 1−MSE/Var(y)). Metrics were averaged across the 5 folds, with standard deviations reported to quantify split-induced variability.

### Structural data and contact graph construction

Three-dimensional protein structures were retrieved from the AlphaFold Protein Structure Database (AFDB, release v6, synchronized with UniProt 2025_03; (Varadi et al. 2024)) using the AlphaFold API (https://alphafold.ebi.ac.uk/api/prediction/). For each protein, the metadata endpoint was queried to obtain the PDB file URL, predicted local distance difference test (pLDDT) confidence scores, and canonical sequence. API-returned sequences were cross-validated against the DMS-derived wild-type sequences at the character level before structure download, providing an additional safeguard against isoform or identifier mismatches.

All 3 structures passed validation: CYP2C9 (AF-P11712-F1-model_v6, 490 residues, global pLDDT 92.94), CYP2C19 (AF-P33261-F1-model_v6, 490 residues, global pLDDT 94.19), and NUDT15 (AF-Q9NV35-F1-model_v6, 164 residues, global pLDDT 92.75). In all 3 cases, model coordinates were inherited from AFDB v4 and unchanged in v6 (v6 affecting only metadata synchronization with UniProt). The fraction of residues with pLDDT ≥70 (confident or higher) was 97.3%, 96.9%, and 92.7%, respectively, indicating that the vast majority of DMS-annotated positions fall within high-confidence regions.

Structures were parsed with Biopython (v1.83), and Cα coordinates were extracted for each residue. Contact graphs were constructed with a Cα-Cα distance threshold of 8 Å, which yielded an average node degree of 9.7 for CYP2C9 and CYP2C19 and 9.1 for NUDT15 (Table 2). Self-loops were excluded from the raw edge set but added explicitly within graph convolution layers where applicable. Euclidean distances were retained as edge attributes for downstream use in distance-aware graph convolutions. Alternative thresholds (6 Å, 10 Å) were also computed and archived for sensitivity analysis.

### Structural feature augmentation

To evaluate whether explicit structural context improves variant effect prediction beyond sequence embeddings alone, we designed a controlled ablation comprising 4 structural feature variants (F6a–F6d) extending the best sequence-only configuration F2. For each protein, the AlphaFold Cα distance matrix was used to identify the *k* = 10 nearest 3D neighbors of every residue (excluding self). From these neighborhoods, we computed 4 categories of structural features per position: (i) local confidence features (2 dimensions: normalized pLDDT at the variant position and a binary disorder flag for pLDDT <70); (ii) neighbor ESM-2 pooling (1,280 dimensions: the mean ESM-2 embedding over the 10 nearest spatial neighbors); (iii) neighbor composition and geometry (33 dimensions: amino acid composition within 8 Å, a radial distance histogram in 1 Å bins from 0 to 10 Å, mean pLDDT of the 10 nearest neighbors, normalized graph degree at the 8 Å cutoff, and per-position pLDDT); and (iv) combinations of the above.

The 4 F6 variants selectively combine these feature categories: F6a (1,322 dim): F2 + local confidence features; F6b (2,600 dim): F2 + neighbor ESM-2 pooling; F6c (1,353 dim): F2 + neighbor composition and geometry; F6d (2,634 dim): F2 + all structural features combined. All F6 variants used the same MLP architecture, optimizer, and 5-fold position-based cross-validation splits as the F1–F5 feature variants (Sections “Model architecture and cross-validation protocol”), enabling direct paired comparison.

### Statistical significance testing

To determine whether observed performance differences between feature variants reflect genuine model improvements rather than fold-to-fold variance, we performed paired significance testing on the 5-fold test-set Pearson correlations. For each variant and dataset, we computed the per-fold difference in Pearson *r* relative to the baseline (Δ*r_i_* = *r*_variant,*i*_−*r*_baseline,*i*_ for *i* = 1, …, 5) and evaluated its statistical significance using 2 complementary tests. First, a paired Wilcoxon signed-rank test assessed whether the median Δ*r* differs from zero under minimal distributional assumptions, appropriate for small-sample paired data. Second, we computed 95% confidence intervals for the mean Δ*r* using paired bootstrap resampling with 10,000 iterations, providing an interval-based complement to the hypothesis test. A variant was considered significantly different from the baseline when the 95% bootstrap CI excluded zero, with the Wilcoxon *P*-value reported alongside as a complementary descriptor; the joint criterion of CI exclusion together with *P* < 0.05 is unattainable at 5 folds and is therefore not used. This follows from the fact that at *n* = 5 folds the minimum achievable Wilcoxon *P*-value is 2/2^5^ = 0.0625, regardless of effect magnitude. This protocol was applied identically to F6 structural variants (Section “Structural feature augmentation yields no detectable improvement over the sequence-only baseline”), the F7 multitask model (Section “Multitask learning yields no detectable improvement and degrades 2 of 4 tasks”), and the F8/F9 GNN models (Section “Contact-graph message passing over frozen embeddings degrades prediction”). Two further considerations bound the interpretation of these tests. Because 34 extension-vs-baseline comparisons are reported (20 for F6, 4 for F7, 10 for F8/F9; the 5 F8-MLP comparisons are a structure-agnostic control rather than a proposed extension and are excluded from this family), inflation of the family-wise error rate is a relevant concern. Multiplicity correction guards against false claims of improvement, and only one comparison of the 34 yielded an interval excluding zero in the positive direction (F6c on NUDT15 stability, Δ*r* = +0.025, 95% CI [+0.005, +0.049]). This is the number of nominal 5% false positives expected from 34 comparisons; the effect is not reproduced by F6c on any other dataset nor by any other F6 variant on that dataset, its magnitude lies below the fold-to-fold standard deviation of F2 on NUDT15 stability (±0.057), and it does not survive a Bonferroni threshold of *α* = 0.05/20 = 0.0025. It is therefore not treated as evidence of a systematic benefit, and a stricter threshold only reinforces the negative conclusion. The intervals that do exclude zero lie entirely in the direction of degradation, and for the graph models the decrements (−0.031 to −0.227 Pearson *r*) exceed the fold-to-fold standard deviation several-fold and are negative in every fold of every dataset, so they are robust to any reasonable family-wise correction; the 2 smaller multitask degradations are reported as detectable under the present design rather than as effects surviving strict correction. All results are conditioned on a single position-level partition (random_state = 42). Position-based assignment removes the dominant source of partition-dependent variation by keeping all variants at a residue together, and every comparison is paired within fold, so partition-induced shifts common to both models cancel in Δ*r*; the absolute correlations reported here are nonetheless specific to this partition, and repetition across multiple partitions would be required to characterize that dependence fully. Accordingly, negative results are stated throughout as the absence of a detectable improvement under the evaluated cross-validation design rather than as evidence of no improvement.

### Multitask learning architecture

To evaluate whether jointly modeling paired DMS phenotypes from the same protein improves variant effect prediction, we constructed a multitask variant (F7) of the F2 baseline. Paired variants were extracted from datasets with overlapping HGVS annotations: CYP2C9 activity and abundance (Amorosi et al. 2021) shared 4,421 variants, and NUDT15 stability (VAMP-seq) and thiopurine sensitivity (Suiter et al. 2020) shared 2,844 variants. For each protein, per-task scores were *z*-score-normalized within each cross-validation fold, using the mean and standard deviation of the training positions only, with validation positions excluded from the calculation. The resulting statistics were applied unchanged to the corresponding validation and test folds, so that no test-fold observation contributes to the scaling applied to it.

The F7 architecture retains the F2 input featurization (1,320-dimensional vector: ESM-2 per-residue embedding of length 1,280 concatenated with 20-dimensional one-hot encodings of the wild-type and mutant amino acids) and extends it with a shared two-layer MLP backbone (1,320 → 512 → 256, ReLU activations, dropout 0.2) followed by 2 task-specific heads (256 → 64 → 1, ReLU, dropout 0.2). The total parameter count is approximately 840,000, roughly 20% larger than the single-task F2 model to keep the comparison approximately capacity-matched per task. During training, both heads were optimized jointly using a weighted-sum loss *L* = *α* · MSE(ŷA, yA) + (1−*α*) · MSE(ŷB, yB) with *α* = 0.5 (equal task weighting). Early stopping was performed on the mean of the 2 per-task validation Pearson correlations. All other hyperparameters, position-based 5-fold cross-validation with random state 42, 20% inner validation split, Adam optimizer with learning rate 1e−3 and weight decay 1e−5, batch size 128, maximum 100 epochs with patience 12, matched the F2 protocol (Section “Model architecture”) to enable direct paired comparison. Single-task F2 references were retrained from scratch on the same paired variant subset and identical CV folds to isolate the multitask effect from any variant-set differences. Statistical significance of Δ*r* was assessed using the protocol described in Section “Statistical significance testing”.

### Graph neural network architectures

To evaluate whether explicit relational modeling of residue–residue contacts yields gains beyond implicit encoding by the ESM-2 language model, we implemented 2 graph neural network (GNN) variants (F8-GCN and F9-GAT) together with a matched structure-agnostic ablation (F8-MLP). Graph convolutional networks (GCN) propagate node features through spectral graph convolution with symmetric normalization, imposing translation-invariance across the graph. Graph Attention Networks (GAT; (Veličković et al. 2018)), in their updated GATv2 formulation (Brody et al. 2022), instead learn edge-specific attention weights, allowing the network to weight structural neighbors differentially based on feature content. Testing both architectures under identical data splits and training regimens enables us to assess whether the performance of graph-based prediction depends on the specific graph operator. We note that more specialized protein-geometric architectures exist (GVP-GNN, EquiformerV2, GearNet), but these were not included here because the primary objective is to assess whether the structural contact graph provides incremental value beyond sequence embeddings, not to identify the state-of-the-art architecture; a systematic comparison with rotation-equivariant architectures is planned for future work as pharmacogene DMS datasets continue to expand.

All 3 models share a two-stage architecture: a node encoder produces per-residue embeddings from the full protein graph in a single forward pass, and a variant-specific prediction head consumes the encoded embedding at the mutated position together with one-hot encodings of the wild-type and mutant amino acids. This design decouples sequence- and structure-level representation learning from mutation-specific inference and enables a direct ablation of the graph component while holding the rest of the pipeline fixed. Graphs were constructed from the AlphaFold-predicted monomeric structures described in Section “Structural data and contact graph construction”. An undirected edge was placed between every pair of residues whose Cα atoms lay within 8 Å, with self-loops added explicitly within convolution layers. This produced 2,374 edges for CYP2C9, 2,366 for CYP2C19, and 748 for NUDT15. Node features at each residue were the concatenation of the ESM-2 per-residue embedding (1,280 dimensions) and the residue's normalized pLDDT score (1 dimension), yielding a 1,281-dimensional input vector per node. Node features are shared across all variants at a given protein: the graph and its features encode protein-level context, while variant-specific information is injected only at the prediction head.

F8-MLP (the structure-agnostic ablation) replaces the graph convolution stack with a position-wise two-layer MLP applied independently to each node (1,281 → hidden → hidden, ReLU, dropout), effectively ignoring the edge index. This control isolates the effect of message passing from any other pipeline difference between F8-GCN/F9-GAT and the F2 baseline (Section “Model architecture”). F8-GCN uses a stack of GCNConv layers with add_self_loops=True, each followed by ReLU and dropout. F9-GAT uses GATv2Conv layers with multihead attention (4 heads, channel-split), ELU activations between layers, and a final single-head layer that projects back to the hidden dimension. All 3 models feed the encoded node embedding at the variant position into a shared 3-layer MLP head (hidden + 40 → 128 → 64 → 1, where the 40 additional inputs are the concatenated wild-type and mutant one-hot encodings).

Hyperparameters (hidden dimension, number of graph convolution layers, and initial learning rate) were selected by grid search on the NUDT15 stability dataset alone, exploring hidden dimensions of 128 and 256, layer counts of 2 and 3, and learning rates of 1 × 10^−3^ and 5 × 10^−4^ under the same position-based 5-fold cross-validation protocol. This yielded a grid of 8 configurations (2 hidden dimensions × 2 layer counts × 2 learning rates) evaluated separately for each of the 3 model classes, 24 model fits in total. The configuration with the highest mean test Pearson correlation was then fixed for all downstream evaluations, resulting in a hidden dimension of 256 with 2 layers and a learning rate of 1 × 10^−3^ for F8-MLP, a hidden dimension of 128 with 3 layers and a learning rate of 1 × 10^−3^ for F8-GCN, and a hidden dimension of 256 with 2 layers and a learning rate of 1 × 10^−3^ for F9-GAT. These settings were then applied across all 5 datasets without per-dataset tuning, in order to avoid optimistic bias from multiple selection. This selection used mean test-fold performance rather than a held-out validation split, which departs from the standard separation between model selection and evaluation and is acknowledged among the methodological boundaries. The direction of the resulting bias is conservative with respect to the conclusions drawn here, since the configuration was chosen to maximize the apparent performance of each model class on the data against which it is subsequently evaluated, and the graph models nonetheless underperformed the matched structure-agnostic control in every comparison.

Training used the Adam optimizer with weight decay 1e−5, mean squared error loss on the raw DMS scores, gradient norm clipping at 1.0, dropout 0.2, batch size 256, and a maximum of 100 epochs with early stopping on validation Pearson correlation (patience = 15). Position-based 5-fold cross-validation, seeds, and inner validation splits were identical to the F2 protocol (Section “Model architecture”) to enable direct paired comparison. Significance of Δ*r* against F2 was assessed as described in Section “Statistical significance testing”.

Position-based cross-validation, in which all variants at a residue position are held in a single fold, eliminates within-position correlation between train and test. However, in a graph-based model, structural context from test positions is necessarily visible to the model during training through the shared graph and the static ESM-2 embedding that encodes local sequence context. This is not a violation of the cross-validation protocol in the classical sense; the labels at test positions are never seen, but it does mean that the model has access to the features of held-out positions via their structural and sequence context. This mirrors the situation in the sequence-only baseline (Section “Model architecture”), where ESM-2 also encodes context from all residues regardless of fold assignment. We therefore interpret the reported test-set correlations as measuring generalization to variants at novel positions when the protein structure and wild-type sequence are fully known, a scenario consistent with the intended deployment of such models for prospective variant interpretation.

### Classification and function-specific detection metrics

To complement the continuous evaluation, the held-out test predictions of the F2 baseline were retained for every variant in all 5 datasets and used to compute metrics aligned with pharmacogenomic use. Variants with a measured score below 0.3 were treated as severe functional loss, matching the boundary that defines the biochemical categories in Section “A general-purpose pathogenicity predictor does not separate biochemically distinct variant categories”; because low predicted scores indicate damage, the ranking score is the negated prediction. Area under the receiver operating characteristic curve (AUROC) and area under the precision–recall curve (AUPRC) were computed within each test fold and averaged, with the positive-class prevalence reported as the AUPRC floor. Precision among the k most damaging-looking variants was computed on the pooled predictions for *k* = 50, 100, and 200, and calibration was summarized by the slope and intercept of the regression of measured on predicted score, for which a perfectly calibrated model gives a slope of one and an intercept of zero.

Whether the models recover function-specific effects rather than only the marginal score of each assay was assessed on the paired variant sets. For each variant measured in both assays of a protein, the difference between the 2 independently trained single-task F2 predictions was compared with the measured assay difference. Three quantities are reported: the correlation between predicted and measured difference; the ability to separate the discordant class defined by a measured absolute difference above 0.3; and the ability to recover the directional categories of Section “A general-purpose pathogenicity predictor does not separate biochemically distinct variant categories”, namely stable-but-dead CYP2C9 variants scored by predicted abundance minus predicted activity, and paradoxical-resistant NUDT15 variants scored by predicted sensitivity minus predicted stability. Because the 2 assays of a protein cover slightly different position sets and therefore receive different fold partitions, these paired analyses are not fold-stratified; confidence intervals are percentile bootstrap intervals over variants with 2,000 resamples. Every prediction entering these analyses is a held-out prediction for its own assay, so no variant is scored by a model trained on its position.

These metrics were computed from a rerun of the F2 configuration under the identical protocol, folds, and seeds described above. The rerun reproduced the mean test Pearson correlations of Table 3 to within 0.017 across the 5 datasets, the residual difference reflecting the different hardware and library environment in which it was executed.

## Results

### Dataset characterization and coverage

The 5 DMS datasets (Table 1) collectively contained 26,198 missense variant measurements distributed across 477 and 486 residue positions in the 2 CYP2C9 assays (97.3 and 99.2% position coverage; 46 and 40 positions with full 19-amino-acid saturation, respectively), 486 residue positions in CYP2C19 (99.2% coverage; 190 positions with full saturation), and 163 residue positions in NUDT15 (100% coverage; 96 and 100 positions with full saturation in the stability and sensitivity assays, respectively). CYP2C19 provided the highest-resolution dataset in terms of both variant count and positional saturation, reflecting the recent nature of the Boyle et al. (2024) data generation. The complete per-position saturation profile is provided in Supplementary Table 3.

**Table 1.** Deep mutational scanning datasets analyzed in this study.

Dataset	Gene (UniProt)	Assay	Reference	*N* variants	*N* positions	Coverage	Fully saturated positions
**CYP2C9 activity**	CYP2C9 (P11712)	Click-seq catalytic	Amorosi et al. 2021	6,142	477	97.3%	46
**CYP2C9 abundance**	CYP2C9 (P11712)	VAMP-seq stability	Amorosi et al. 2021	6,370	486	99.2%	40
**CYP2C19 abundance**	CYP2C19 (P33261)	VAMP-seq stability	Boyle et al. 2024	7,830	486	99.2%	190
**NUDT15 stability**	NUDT15 (Q9NV35)	VAMP-seq stability	Suiter et al. 2020	2,922	163	100%	96
**NUDT15 sensitivity**	NUDT15 (Q9NV35)	Thiopurine sensitivity	Suiter et al. 2020	2,934	163	100%	100

*N* variants refers to missense variants retained after filtering. Fully saturated positions indicate residues at which all 19 possible amino acid substitutions have been measured. Coverage is the percentage of residue positions with at least one measured substitution. All datasets were validated against UniProt canonical sequences with 100% wild-type amino acid concordance (Section “Variant parsing and wild-type validation”).

**Table 2.** AlphaFold structural data properties and DMS position coverage.

Protein	AlphaFold model	*N* residues	Mean pLDDT	% residues pLDDT ≥70	Cα edges (<8 Å)	Avg. degree	DMS positions in structure
**CYP2C9**	AF-P11712-F1-v6	490	93.0	97.3%	2,374	9.7	486/486 (100%)
**CYP2C19**	AF-P33261-F1-v6	490	94.2	96.9%	2,366	9.7	486/486 (100%)
**NUDT15**	AF-Q9NV35-F1-v6	164	92.7	92.7%	748	9.1	163/163 (100%)

Mean pLDDT at DMS-annotated positions was 92.9–94.2 across proteins, and 2.5–6.7% of positions fell below the pLDDT ≥70 threshold (CYP2C9: 2.5–2.7%; CYP2C19: 3.1%; NUDT15: 6.7%). These low-confidence positions were retained in the graph, with pLDDT included as a per-node feature rather than used as a filtering criterion.

### Functional heterogeneity in CYP2C9 and NUDT15

Functional heterogeneity between paired biochemical assays was examined across the 5 datasets to establish the empirical basis for distinguishing protein-level from substrate-level variant effects. The joint distributions of activity vs abundance for CYP2C9 and of thiopurine sensitivity vs stability for NUDT15 revealed substantial decoupling in both enzymes, with magnitudes consistent with a biologically meaningful rather than artefactual origin. The complete exploratory characterization is summarized in Fig. 1.

**Fig. 1.**
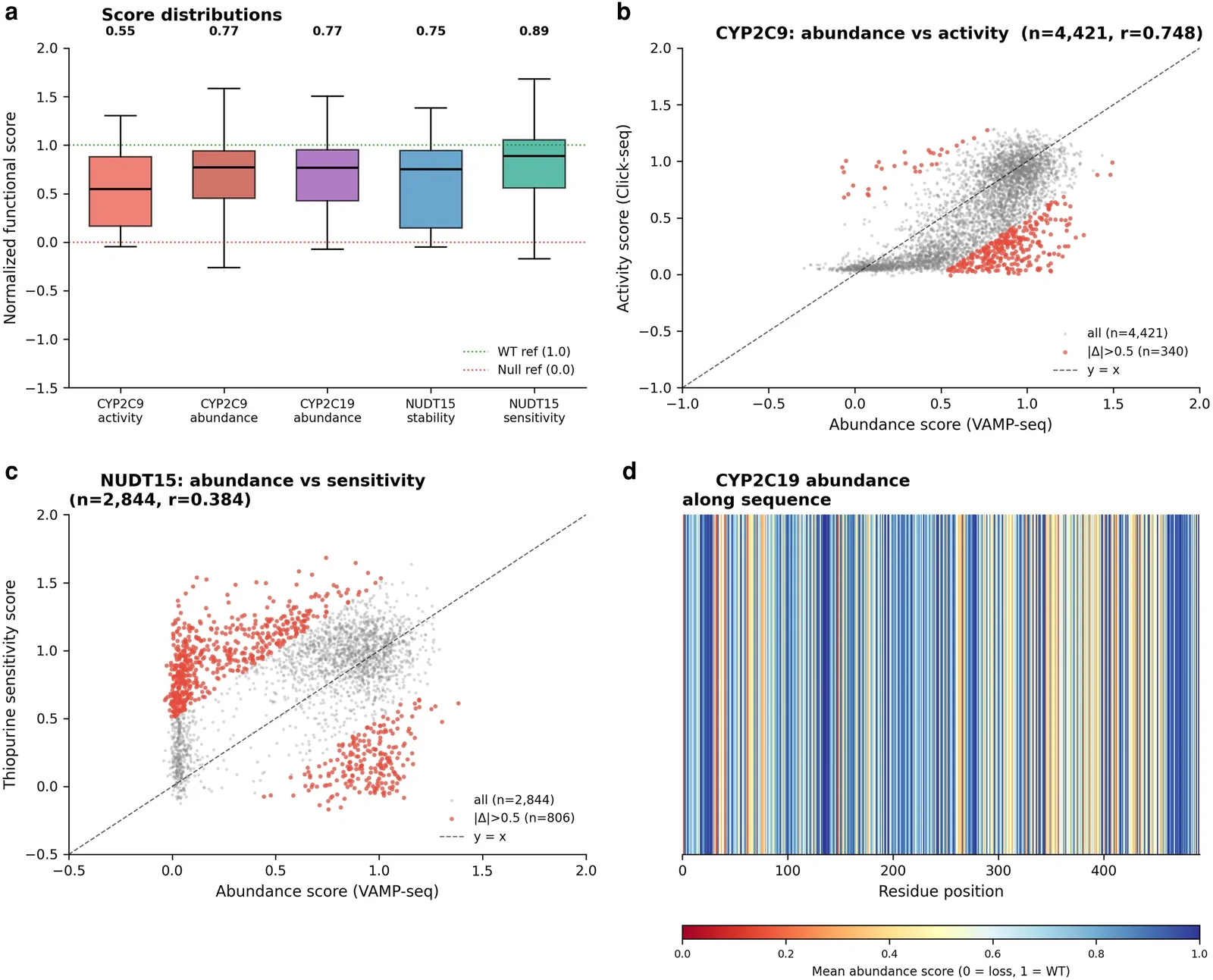
Exploratory characterization of pharmacogene DMS datasets. a) Distribution of normalized functional scores for all 5 datasets. Bold numbers above each box indicate the median; the upper and lower reference lines mark the expected wild-type (1.0) and null (0.0) values, respectively, as labeled in the legend. The CYP2C9 activity distribution has a median of 0.55, substantially lower than the other 4 (medians 0.75–0.89), consistent with the Click-seq assay being more stringent in detecting catalytic impairment than the VAMP-seq-based abundance assays. b) Joint distribution of CYP2C9 abundance (*x*-axis, VAMP-seq) and activity (*y*-axis, Click-seq) for *n* = 4,421 variants measured in both assays (Pearson *r* = 0.748). Highlighted points indicate the 340 variants with |activity − abundance| > 0.5. This cutoff is more stringent than the 0.3 threshold used for the statistics reported in the text, and is used in the figure only for legibility, since highlighting all 1,236 variants above the 0.3 threshold would obscure the underlying joint distribution. The full threshold sweep is given in Supplementary Table 1. The dashed diagonal denotes perfect concordance. c) Joint distribution of NUDT15 abundance (*x*-axis) and thiopurine sensitivity (*y*-axis) for *n* = 2,844 variants (Pearson *r* = 0.384). Highlighted points indicate the 806 variants with |sensitivity − abundance| > 0.5, again more stringent than the 0.3 threshold used in the text and applied here for the same reason of legibility. d) Position-averaged CYP2C19 abundance scores along the 490-residue sequence, shaded on a continuous scale from complete loss of function (score 0) to wild-type preservation (score 1). Alternating high- and low-function regions correspond to known structural motifs of the CYP fold.

The score distributions of all 5 missense variant sets (Fig. 1a) were normalized such that wild-type ≈ 1.0 and nonsense ≈ 0.0. The CYP2C9 activity distribution has a median of 0.55, substantially lower than the other 4 distributions (medians 0.75–0.89), consistent with the Click-seq assay being more stringent in detecting catalytic impairment than the VAMP-seq-based abundance assays.

For CYP2C9, the joint distribution of activity and abundance scores was analyzed across the 4,421 variants measured in both assays (Fig. 1b). The Pearson correlation coefficient (*r* = 0.748) closely matched the value reported by Boyle et al. (2024; *r* = 0.76), providing an independent cross-validation of the data ingestion and parsing pipeline. Notably, 1,236 variants (28% of the joint set) exhibited absolute differences greater than 0.3 normalized units between the 2 assays, and the vast majority of these (1,113 of 1,236, or 90%) showed abundance substantially higher than activity, indicative of a stable but catalytically inactive protein. The fraction classified as function-specific varies smoothly with the cutoff, from 69.2% at a threshold of 0.10 to 4.3% at 0.60, with no discontinuity that would indicate sensitivity to the particular value chosen (Supplementary Table 1). The predominance of the stable-but-catalytically-impaired direction is likewise preserved, rising from 80% of discordant variants at the loosest cutoff to a plateau near 91% at cutoffs of 0.35 and above. The 0.3 threshold used for the headline statistic was selected for consistency with the 0.3 and 0.7 score boundaries that define the biochemical categories in Section “A general-purpose pathogenicity predictor does not separate biochemically distinct variant categories”. The top 10 variants by absolute activity–abundance discordance localized to positions Ser365, Glu300, Ala297, Val113, and Cys435, which were independently identified by Boyle et al. as residues within the substrate-binding pocket and relevant to catalytic specificity for drugs including warfarin, flurbiprofen, celecoxib, and phenytoin. This concordance between the data-driven classification and the published structural biochemistry of CYP2C9 supports the biological validity of the function-specific variant category. The complete list of 4,421 paired CYP2C9 variants, including the 1,236 function-specific variants (|activity − abundance| > 0.3) and their AlphaMissense annotations, is provided in Supplementary Table 4.

The analogous analysis for NUDT15 compared the paired abundance and sensitivity scores across 2,844 variants (Fig. 1c). The Pearson correlation was substantially lower (*r* = 0.384), and 1,364 variants (48% of the joint set) exhibited absolute differences greater than 0.3 normalized units, indicating that for NUDT15 the effect of a variant on protein stability is only weakly predictive of its effect on thiopurine sensitivity. The corresponding sweep yields discordant fractions from 78.3 to 21.1% across the same threshold range (Supplementary Table 1). At every cutoff examined, the NUDT15 discordant fraction exceeds that of CYP2C9, so the difference between the 2 proteins does not depend on where the threshold is placed. Given that NUDT15 variants contribute to thiopurine-induced myelosuppression in patients receiving azathioprine or 6-mercaptopurine for inflammatory bowel disease, acute lymphoblastic leukemia, or transplant immunosuppression (Maillard et al. 2024), this functional decoupling has direct clinical relevance. The complete list of 2,844 paired NUDT15 variants, including the 1,364 substrate-specific variants (|sensitivity − abundance| > 0.3) and their AlphaMissense annotations, is provided in Supplementary Table 5.

A position-averaged view of CYP2C19 abundance scores along the 490-residue protein is provided in Fig. 1d, colored on a blue (preserved)-to-red (disrupted) gradient. The visualization reveals conserved high-function regions (heme-binding motif, transmembrane anchor) interspersed with loss-of-function-prone regions corresponding to known substrate channels and active site residues, consistent with the canonical CYP folding topology.

### Feature ablation of the supervised baseline

The contribution of sequence context, amino acid identity, spatial neighborhood, and global statistics to variant effect prediction was evaluated by comparing 5 feature representations under the position-based 5-fold cross-validation protocol. Feature variant F2, which concatenates the ESM-2 per-residue embedding with one-hot encodings of the wild-type and mutant amino acid identities, achieved the highest mean Pearson correlation on every dataset, with peak performance on CYP2C19 abundance (*r* = 0.720 ± 0.025), followed by CYP2C9 abundance (*r* = 0.675 ± 0.031), CYP2C9 activity (*r* = 0.667 ± 0.033), NUDT15 sensitivity (*r* = 0.659 ± 0.031), and NUDT15 stability (*r* = 0.537 ± 0.057). The complete test-set correlations for all 5 feature variants across all 5 datasets are summarized in Table 3.

**Table 3.** Five-fold position-based cross-validation results (test Pearson *r*, mean ± SD across folds) for 5 feature representations.

Dataset	F1 (pos)	F2 (pos + onehot)	F3 (window)	F4 (delta)	F5 (full)
CYP2C9 activity	0.583 ± 0.047	**0.667** ± **0.033**	0.590 ± 0.030	0.647 ± 0.028	0.615 ± 0.037
CYP2C9 abundance	0.531 ± 0.040	**0.675** ± **0.031**	0.604 ± 0.054	0.637 ± 0.062	0.637 ± 0.053
CYP2C19 abundance	0.583 ± 0.026	**0.720** ± **0.025**	0.629 ± 0.018	0.684 ± 0.036	0.656 ± 0.013
NUDT15 sensitivity	0.629 ± 0.021	**0.659** ± **0.031**	0.504 ± 0.088	0.655 ± 0.024	0.570 ± 0.049
NUDT15 stability	0.495 ± 0.029	**0.537** ± **0.057**	0.427 ± 0.123	0.533 ± 0.040	0.451 ± 0.098

Bold values indicate the highest mean Pearson *r* per dataset. F1 = position embedding only (1,280 dim); F2 = F1 + wild-type/mutant one-hot (1,320 dim); F3 = ±2-residue window + one-hot (6,440 dim); F4 = position + delta from mean + mean + one-hot (3,880 dim); F5 = all components combined (9,000 dim). MLP hidden-layer widths adapt automatically to input dimension (256/64 for ≤2,000; 512/128 otherwise), so feature content and model capacity co-vary across variants and their separate contributions cannot be resolved from this comparison alone. All models were trained with deterministic CUDA settings, seed 42 for fold assignment, and fold-specific seeds for model initialization.

F2 was selected as the primary supervised baseline on empirical grounds rather than a priori, since it achieved the highest mean test Pearson correlation on all 5 datasets under a protocol in which every feature variant was trained on identical folds. All subsequent architectural extensions (F6, F7, F8, F9) were built on this configuration, so the selection was fixed before any extension was evaluated. This selection used mean test-fold performance across the 5 datasets, as did the graph neural network hyperparameter search described in Section “Graph neural network architectures”; the implications of both are addressed among the methodological boundaries. Spearman rank correlations computed on the same folds are reported for all 25 feature–dataset combinations in Supplementary Table 6. The ranking is unchanged: F2 achieves the highest mean Spearman correlation on every dataset, so the selection of F2 as the primary baseline does not depend on the choice of correlation coefficient. The 2 coefficients diverge most on NUDT15 sensitivity, where F2 reaches *ρ* = 0.580 against *r* = 0.668, indicating that the model's rank ordering on the one substrate-dependent assay is weaker than its linear agreement; the same asymmetry appears independently in the top-k prioritization results of Section “The baseline identifies damaging variants but not the dimension along which they are damaging”. Three key observations emerge from these results. The consistent superiority of F2 over all higher-dimensional alternatives is nontrivial, because the intuition that richer feature representations should yield better predictions is not supported in this regime. The comparison does not isolate the cause. Hidden-layer width was tied to input dimensionality (Section “Model architecture”), so the higher-dimensional variants differ from F2 in both the information they encode and the capacity of the network consuming it; the first linear layer alone comprises approximately 0.34 million parameters under F2 and approximately 3.3 million under F3, fitted to the same few thousand labelled variants. Whether the higher-dimensional representations carry no additional usable signal, or carry signal that this configuration cannot exploit at this sample size, cannot be determined from these results. The absolute performance achieved by F2 (*r* = 0.537–0.720) is comparable to published benchmarks for comparable pharmacogene DMS prediction tasks: Hubble.2D6 reports *R*^2^ ≈ 0.475 for CYP2D6 (Mcinnes et al. 2020), corresponding to *r* ≈ 0.69, and the Van Der Lee et al. (2021) neural network achieves approximately 79% variance explanation on CYP2D6 tamoxifen metabolism with supervised training on patient-derived metabolic ratios. Beyond these absolute values, fold-to-fold standard deviations remained modest for the larger datasets (±0.025 to ±0.033 for CYP2C19 and CYP2C9 under F2) but inflated for NUDT15 due to the smaller number of positions available for splitting (163 total, approximately 33 per fold), a limitation intrinsic to the protein size rather than to the methodology.

### Implications for architectural design

The feature ablation results provide a starting point for evaluating architectural extensions. Feature variants F3 and F5 did not improve over F2, but for the reasons given above, this comparison cannot separate feature content from model capacity, so it establishes only that the particular combinations of representation and network evaluated here did not outperform the simplest one. Whether local spatial context and global sequence statistics carry usable signal for this task therefore remains open, and the extensions evaluated in the remainder of this study approach that question through mechanisms other than input concatenation. These feature-level results motivate the subsequent evaluation of 3 explicit architectural extensions: structural feature augmentation from AlphaFold contact graphs (Section “Structural feature augmentation yields no detectable improvement over the sequence-only baseline”), multitask joint learning across paired phenotypic assays (Section “Multitask learning yields no detectable improvement and degrades 2 of 4 tasks”), and graph neural network modeling of residue–residue contacts (Section “Contact-graph message passing over frozen embeddings degrades prediction”).

### Comparison with zero-shot and pretrained baselines

To contextualize the supervised baseline against established alternatives, F2 was compared with 2 widely adopted models. The first comparator was the ESM1v ensemble zero-shot scoring method (Meier et al. 2021), computed by averaging the masked-language-model log-likelihood ratios from 5 independently trained ESM1v 650 M models (UR90S). Because the masked-language-model log-likelihood ratio is higher for substitutions the model regards as tolerated, ESM1v scores correlate positively with the DMS functional scores and required no sign adjustment; the ESM1v values reported below are raw Pearson correlations, which were positive in every case. These likelihood-ratio values were used here as a standalone zero-shot predictor rather than as input features to the supervised model; combining them with the F2 representation is a natural extension that was not attempted in this study. The second comparator was AlphaMissense (Cheng et al. 2023), retrieved from the public release of precomputed pathogenicity scores for all possible missense substitutions in the human proteome. AlphaMissense is included here as a representative general-purpose pathogenicity predictor rather than as a model intended for the tasks evaluated in this study, since it is trained to predict clinical pathogenicity rather than substrate-specific activity, folded abundance, or drug-response phenotypes, and any discrepancy between its scores and the DMS measurements therefore reflects in part a mismatch between its training objective and the evaluation target rather than a deficiency of the model on the task for which it was designed. The AlphaMissense database achieved 100% coverage of the variant set analyzed here, reflecting its exhaustive annotation of canonical UniProt sequences. Because AlphaMissense raw scores range from 0 (benign) to 1 (pathogenic), the correlation with DMS functional scores is expected to be negative, and absolute correlation values are therefore reported to facilitate direct comparison. The side-by-side performance of all 3 approaches across the 5 datasets is summarized in Table 4.

**Table 4.** Comparison of the supervised F2 baseline with the zero-shot ESM1v ensemble and AlphaMissense pretrained predictions.

Dataset	ESM1v (zero-shot)	AlphaMissense	F2 supervised (ours)	Best margin over baselines
CYP2C9 activity	0.656	0.631	**0.667 ± 0.033**	+0.011
CYP2C9 abundance	0.625	0.613	**0.675 ± 0.031**	+0.050
CYP2C19 abundance	0.686	0.688	**0.720 ± 0.025**	+0.032
NUDT15 stability	0.479	0.524	**0.537 ± 0.057**	+0.013
NUDT15 sensitivity	**0.664**	0.634	0.659 ± 0.031	−0.005

All values are test-set Pearson correlation. ESM1v log-likelihood ratios correlate positively with DMS function and are reported unadjusted. For AlphaMissense, raw Pearson *r* is negative as expected, since pathogenicity inversely correlates with DMS function, and the absolute value is reported for comparability.

Bold indicates the best performer per row. The best margin over baselines is the difference between F2 and the stronger of the 2 baselines (ESM1v or AlphaMissense).

Three observations emerge from this comparison. AlphaMissense, despite its broad success on general pathogenicity benchmarks, was consistently outperformed by ESM1v zero-shot on CYP2C9 (activity: 0.631 vs 0.656; abundance: 0.613 vs 0.625) and was essentially tied on CYP2C19 (0.688 vs 0.686), and this inversion of the typical ranking reflects the fact that AlphaMissense is optimized for general clinical pathogenicity, which correlates with protein-level folding disruption, whereas the Click-seq and VAMP-seq assays used here capture biochemically distinct quantities. On the single dataset where AlphaMissense led zero-shot ESM1v (NUDT15 stability, 0.524 vs 0.479), the advantage did not translate into outperforming the supervised F2 baseline, a pattern that underscores that a generic pathogenicity predictor, however powerful, is not a substitute for pharmacogene-specific modeling when the downstream clinical question is enzymatic function rather than disease causality.

The supervised gain over the stronger of the 2 baselines was narrow across all datasets (+0.011 to +0.050), and on NUDT15 sensitivity the zero-shot ESM1v ensemble marginally exceeded F2 (−0.005), indicating that for the sequence-only representation evaluated here the benefit of supervision was small relative to what was achievable without labels. The NUDT15 sensitivity result is mechanistically informative beyond this aggregate pattern: ESM1v zero-shot reached 0.664 on a task (thiopurine sensitivity) that explicitly depends on substrate-specific enzymatic function, while F2 trained on the same data reached only 0.659 ± 0.031, and because ESM1v has no exposure to thiopurine chemistry during pretraining, its performance on this task must be attributable entirely to the conservation signal at residues relevant to NUDT15 folding and active-site architecture. The inability of F2 to systematically exceed this signal, despite direct supervision on the DMS target, suggests that substrate-specific function was not readily recoverable from the wild-type sequence embeddings used here, although this does not exclude the possibility that other sequence-derived representations, such as mutant-sequence embeddings or likelihood-ratio features, would perform differently.

Taken together, these results show that the 3 sequence-based approaches evaluated here, namely the ESM1v zero-shot ensemble, AlphaMissense, and the supervised F2 baseline, reach similar absolute performance on these pharmacogene DMS tasks, with differences measured in a few percentage points of Pearson *r*. This comparison is not an exhaustive survey of sequence-based variant effect prediction, and approaches not evaluated here, including unsupervised generative models such as EVE, likelihood-ratio features from more recent protein language models, mutant-sequence or delta embeddings, lightweight fine-tuning of the language model itself, and non-neural supervised methods, may behave differently. Whether the architectural extensions examined in this study improve upon the F2 baseline is addressed in Sections “Structural feature augmentation yields no detectable improvement over the sequence-only baseline, Multitask learning yields no detectable improvement and degrades 2 of 4 tasks and Contact-graph message passing over frozen embeddings degrades prediction”.

### A general-purpose pathogenicity predictor does not separate biochemically distinct variant categories

To examine how a general-purpose pathogenicity predictor behaves across the biochemical dimensions resolved by the paired assays, the CYP2C9 and NUDT15 joint-assay datasets were stratified into biochemically defined categories and AlphaMissense scores were compared across them. The analysis is framed as a test of alignment between a clinical pathogenicity objective and pharmacogene-specific functional readouts rather than as an evaluation of AlphaMissense on the task for which it was trained. For CYP2C9, variants were classified by joint activity–abundance thresholds into 4 groups: concordant wild-type-like (both scores >0.7), concordant loss-of-function (both scores <0.3), stable-but-dead (abundance >0.7, activity <0.3), and the rare unstable-but-active (abundance <0.3, activity >0.7). For NUDT15, the analogous abundance–sensitivity stratification yielded concordant-resistant (both >0.7), concordant-sensitive (abundance <0.3, sensitivity <0.5), thiopurine-sensitive-stable (abundance >0.7, sensitivity <0.5), and paradoxical-resistant (abundance <0.3, sensitivity >0.8). The 0.3 and 0.7 boundaries follow from the normalization of the assays, in which nonsense variants score near 0 and wild-type near 1, so that these values divide the scale into a lower band, a middle band, and an upper band without reference to the empirical distribution of any one dataset; their robustness is examined below and in Supplementary Table 2. The sensitivity axis uses 0.5 and 0.8 in place of 0.3 and 0.7 because the NUDT15 sensitivity distribution is shifted toward the tolerant end relative to the CYP2C9 activity assay (Fig. 1a), so that a 0.3 cutoff would leave too few variants to populate the sensitive categories and a 0.7 cutoff would admit too many into the resistant one; the 0.8 boundary is varied from 0.70 to 0.90 in Supplementary Table 2. AlphaMissense scores were discretized at the thresholds recommended by its authors, 0.34 for likely benign and 0.564 for likely pathogenic, which were calibrated on ClinVar to yield approximately 90% precision in each direction, rather than at cutoffs derived from the present data. The number of variants, median AlphaMissense score, and discrete classification (benign, ambiguous, or pathogenic) for each category are reported in Table 5, and the corresponding score distributions and scatter plots are shown in Fig. 2. The robustness of these category definitions was assessed by varying the joint cutoffs across 5 paired combinations per protein (Supplementary Table 2). For NUDT15, the paradoxical-resistant category is largely insensitive to the definition: across cutoffs, the median AlphaMissense score remains between 0.671 and 0.700 and the proportion classified as pathogenic between 61.5 and 63.8%, while the category size falls from 338 to 115 variants. For CYP2C9, the stable-but-dead category behaves differently. As the definition tightens, the median AlphaMissense score rises from 0.513 to 0.790, and the proportion scored as likely benign falls from 29.1 to 7.6%, indicating that AlphaMissense scores the most extreme stable-but-dead variants more severely. The existence of a benign-scored subset despite catalytic inactivation therefore holds at every cutoff examined, but its magnitude depends on how stringently the category is defined, and the 38 variants identified below should be read as the subset arising under one particular definition rather than as a fixed count.

**Fig. 2.**
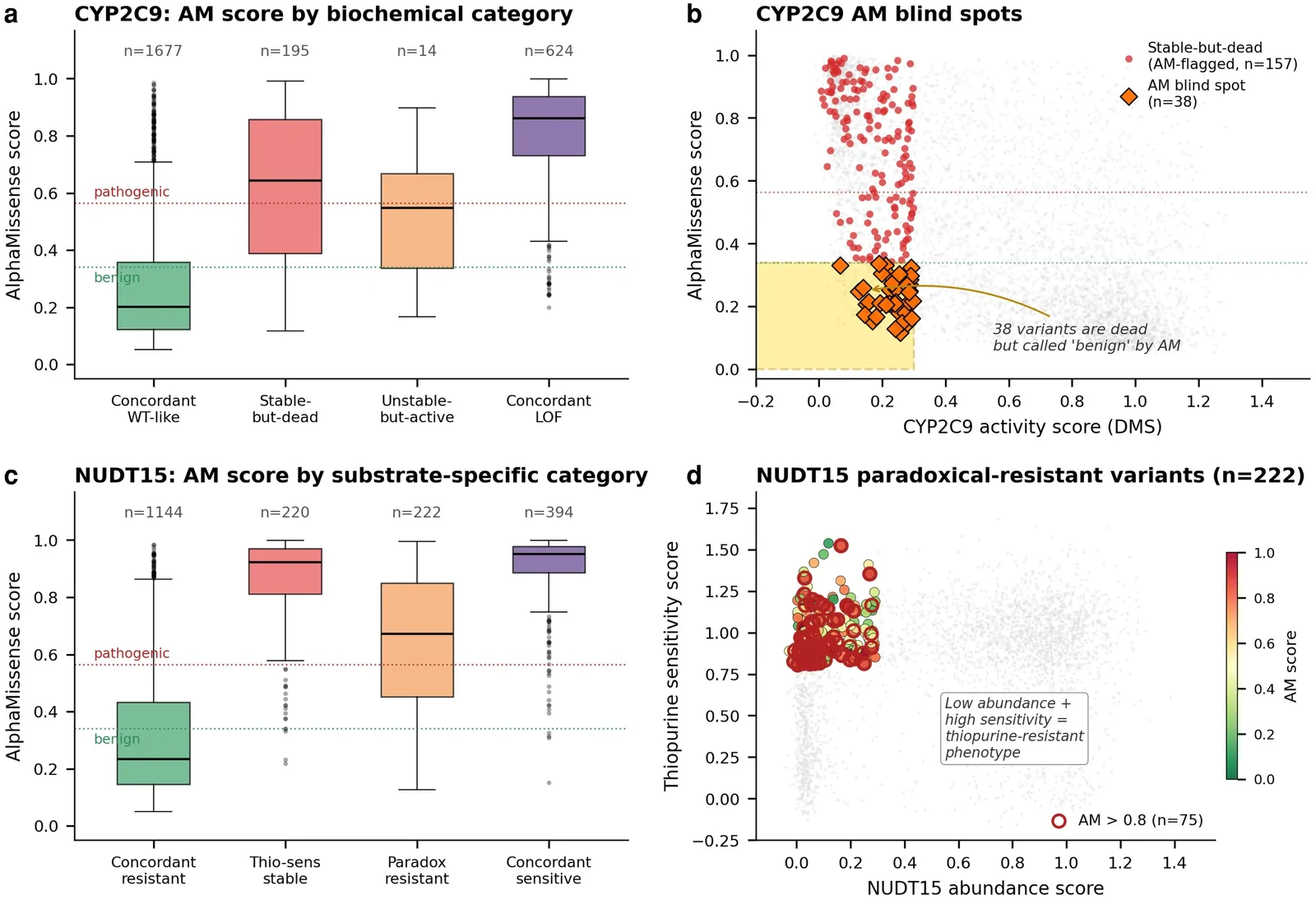
AlphaMissense scores do not separate biochemically distinct variant categories. a) Distribution of AlphaMissense pathogenicity scores across 4 CYP2C9 variant categories defined by joint activity–abundance thresholds. Dotted lines indicate AlphaMissense classification thresholds (0.34 benign, 0.564 pathogenic). The stable-but-dead median (0.64) lies between concordant-LOF (0.86) and concordant-WT (0.20), indicating that AlphaMissense scores do not separate variants that are catalytically inactive but structurally preserved. Mann–Whitney *U* test: stable-but-dead vs concordant-LOF, *P* = 4.15 × 10^−20^; vs concordant-WT, *P* = 4.28 × 10^−57^. b) Scatter plot of all CYP2C9 joint-assay variants, with stable-but-dead variants highlighted (*n* = 195). The shaded region marks the zone of maximal divergence between the AlphaMissense score and the activity measurement: 38 of 195 stable-but-dead variants (19%) fall below the benign threshold despite exhibiting activity <0.3. c) Distribution of AlphaMissense scores across 4 NUDT15 categories. The paradoxical-resistant category (*n* = 222) has a median AlphaMissense score of 0.67 with 63% classified as pathogenic, despite these variants retaining wild-type-like scores in the thiopurine sensitivity assay. d) Scatter plot of NUDT15 abundance vs thiopurine sensitivity, with paradoxical-resistant variants shaded by AlphaMissense score.

**Table 5.** AlphaMissense score distributions across biochemically defined variant categories in CYP2C9 and NUDT15.

Protein	Category	*N*	AM median	Benign	Ambiguous	Pathogenic
CYP2C9	Concordant WT-like	1,677	0.20	1,224 (73%)	247 (15%)	206 (12%)
CYP2C9	**Stable-but-dead**	**195**	**0**.**64**	**38 (19%)**	**47 (24%)**	**110 (56%)**
CYP2C9	Unstable-but-active	14	0.55	4	4	6
CYP2C9	Concordant LOF	624	0.86	19 (3%)	45 (7%)	560 (90%)
NUDT15	Concordant-resistant	1,144	0.23	754 (66%)	199 (17%)	191 (17%)
NUDT15	Thiopurine-sensitive-stable	220	0.92	4 (2%)	15 (7%)	201 (91%)
NUDT15	Concordant-sensitive	394	0.95	4 (1%)	11 (3%)	379 (96%)
NUDT15	**Paradoxical-resistant**	**222**	**0**.**67**	**31 (14%)**	**51 (23%)**	**140 (63%)**

Bold rows highlight categories in which AlphaMissense scores align least closely with the assayed biochemistry.

Proportions in parentheses are calculated within each category. Bold rows highlight categories in which AlphaMissense scores align least closely with the assayed biochemistry. For CYP2C9, 56% of stable-but-dead variants are scored as pathogenic; for NUDT15, paradoxical-resistant variants receive a median AlphaMissense score of 0.67 despite retaining wild-type-like scores in the thiopurine sensitivity assay. Because AlphaMissense is trained on clinical pathogenicity rather than on these assay-specific readouts, these patterns are reported as objective mismatch rather than as classification error.

The extent of this divergence is illustrated by several statistical comparisons between and within the defined categories. For CYP2C9, although stable-but-dead and concordant-LOF variants are both catalytically compromised, their AlphaMissense medians differ substantially (0.64 vs 0.86; Mann–Whitney U = 34,357, *P* = 4.15 × 10^−20^; Fig. 2a), meaning that AlphaMissense assigns a significantly lower pathogenicity score to variants that preserve protein abundance despite their total loss of catalytic activity. A complementary comparison between stable-but-dead and concordant-WT variants shows that although the 2 groups are separated at the aggregate level (medians 0.64 vs 0.20; U = 277,278, *P* = 4.28 × 10^−57^), 38 of the 195 stable-but-dead variants (19%) nonetheless receive a “likely benign” classification, as visualized by the shaded region in Fig. 2b. These 38 variants, concentrated at residues Asn202, Asp188, Ile169, Glu203, Thr392, Lys241, Leu170, Leu197, Leu29, and Cys175, would be scored as tolerated by AlphaMissense despite showing complete catalytic inactivation in the Click-seq assay, and they therefore constitute a candidate set in which a general pathogenicity score and an assay-specific functional measurement diverge, although establishing whether that divergence carries clinical consequence would require patient-level pharmacogenomic evidence that is not available here; the complete annotation of all 195 stable-but-dead variants, including the 38 AlphaMissense-benign subset and per-variant scores, is provided in Supplementary Table 4.

A parallel pattern emerges for NUDT15 (Fig. 2c and d). AlphaMissense separates thiopurine-sensitive-stable from concordant-resistant variants well (medians 0.92 vs 0.23; *P* = 4.55 × 10^−98^), correctly flagging 201 of the 220 thiopurine-sensitive-stable variants (91%) as likely pathogenic, yet it fails to distinguish thiopurine-sensitive-stable from concordant-sensitive (medians 0.92 vs 0.95; *P* = 7.54 × 10^−5^, effect size small despite significance), indicating that AlphaMissense scores do not distinguish whether a variant is deleterious through loss of protein integrity or through substrate-specific sensitivity, which is the distinction relevant to thiopurine dosing. The largest divergence concerns the paradoxical-resistant category of 222 variants, which destabilize the protein (abundance < 0.3) yet paradoxically preserve or enhance thiopurine metabolism (sensitivity >0.8). AlphaMissense assigns these variants a median score of 0.67 with 63% labeled pathogenic, a direction of scoring that runs opposite to the measured thiopurine phenotype, since reduced thiopurine sensitivity would if anything be expected to lower rather than raise toxicity risk; whether this discordance would translate into an incorrect clinical inference cannot be determined from the present data and would require patient-level pharmacogenomic evidence. The complete annotation of all 222 paradoxical-resistant variants, including per-variant AlphaMissense scores and classifications, is provided in Supplementary Table 5.

Beyond these between-category comparisons, the intra-category correlation between AlphaMissense score and the primary functional readout (activity for CYP2C9, sensitivity for NUDT15) is weak to moderate in every category (|*r*| = 0.09 to 0.44), indicating that even within a biochemically homogeneous group AlphaMissense does not act as a linear predictor of the quantitative functional readout. AlphaMissense is therefore better understood as a binary pathogenicity classifier rather than as a substitute for pharmacogene-specific functional prediction.

Taken together, these observations indicate that a predictor trained on clinical pathogenicity does not resolve the mechanism by which a pharmacogene variant is damaging, whether through a folding defect, a substrate-specificity change, or catalytic inactivation, and that this mechanism is the distinction on which pharmacogene interpretation depends. This is a statement about objective mismatch rather than about model quality, since AlphaMissense was neither designed nor trained to make that distinction. Whether multitask output heads trained jointly on paired assays can recover this distinction from data alone, without explicit substrate conditioning, is addressed in Section “Multitask learning yields no detectable improvement and degrades 2 of 4 tasks”.

### Structural feature augmentation yields no detectable improvement over the sequence-only baseline

To test whether explicit three-dimensional context derived from AlphaFold structures provides predictive value beyond what is already encoded in ESM-2 sequence embeddings, 4 structural feature augmentations of the best sequence-only configuration F2 were evaluated (Section “Structural feature augmentation”): local confidence features (F6a), neighbor ESM-2 pooling (F6b), neighbor composition and geometry (F6c), and their full union (F6d). All 4 variants were trained under the identical 5-fold position-based cross-validation protocol and MLP architecture used for F1–F5 (Table 3), enabling direct paired comparison.

Across the 5 DMS datasets and 4 structural variants, no combination produced a systematic improvement over F2. Paired bootstrap 95% confidence intervals for Δ*r* = *r*_F6_−*r*_F2_ included zero in 17 of the 20 comparisons, and Wilcoxon signed-rank tests yielded *P* > 0.1 throughout. Of the 3 intervals that excluded 0, 2 were negative (CYP2C9 activity with F6a and F6c) and one positive: NUDT15 stability with F6c (Δ*r* = +0.025, 95% CI [+0.005, +0.049]). This single positive result is isolated, is not reproduced by F6c on any other dataset nor by any other F6 variant on NUDT15 stability, and falls below the fold-to-fold standard deviation of F2 on that dataset; it is treated as a multiple-comparisons artifact for the reasons set out in Section “Statistical significance testing”. In the opposite direction, F6 variants underperformed F2 in 14 of the 20 combinations, most consistently for CYP2C9 activity, where every F6 variant produced a negative Δ*r*. The complete paired significance testing of all F6 variant–dataset combinations against F2 is summarized in Table 6, and illustrated in Fig. 3b.

**Fig. 3.**
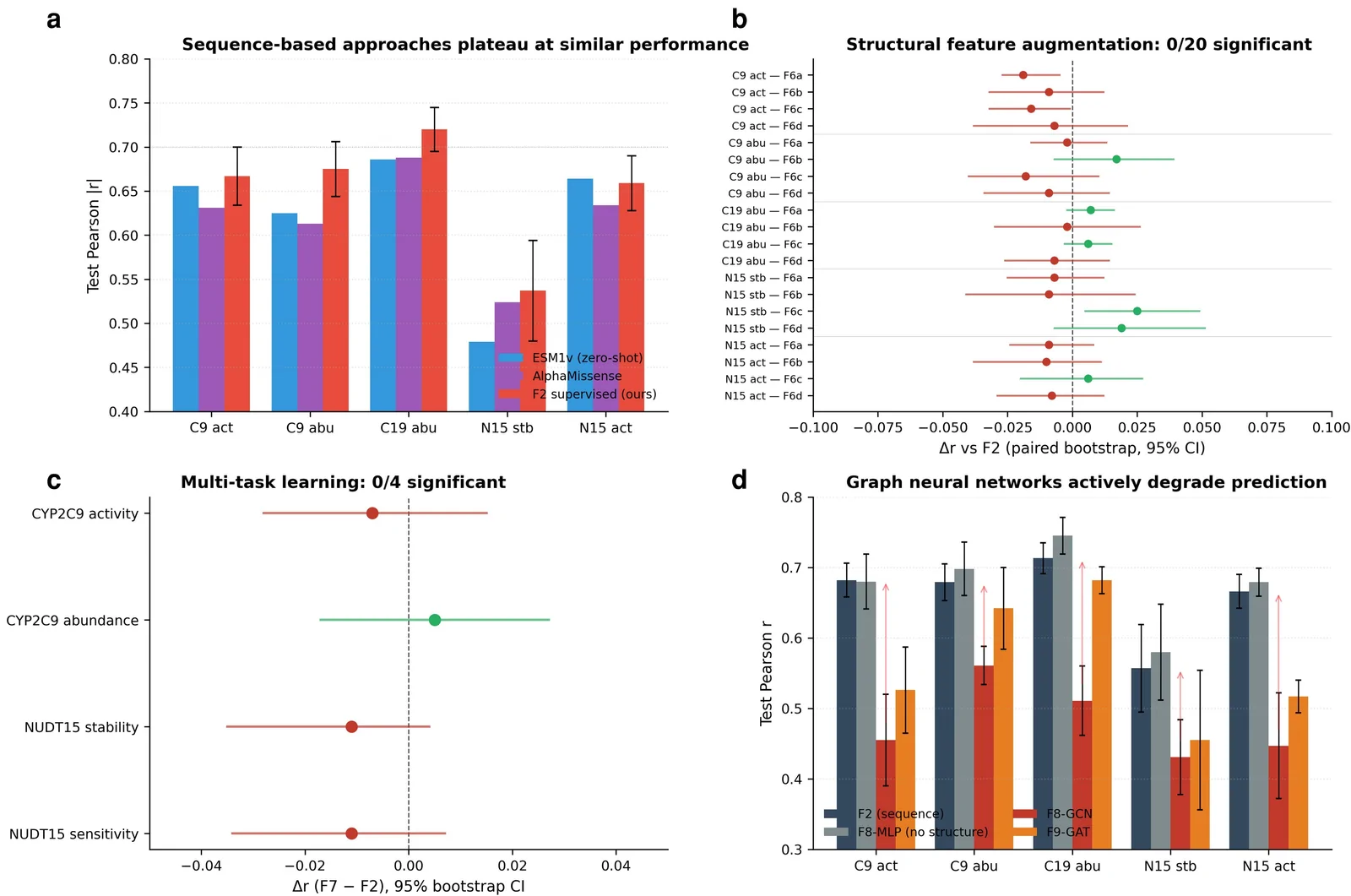
Systematic benchmark of architectural extensions against the sequence-only F2 baseline. a) Test Pearson |*r*| for F2, the zero-shot ESM1v ensemble, and AlphaMissense across the 5 DMS datasets, as labeled in the legend (Table 4). F2 error bars show fold-to-fold standard deviation. All 3 sequence-based approaches plateau at similar performance, with F2 marginally exceeding the baselines in 4 of 5 datasets and the ESM1v zero-shot ensemble slightly exceeding F2 on NUDT15 sensitivity. b) Forest plot of Δ*r* = *r*_F6_−*r*_F2_ for all 20 F6 structural feature variant–dataset combinations (Table 6), with 95% paired bootstrap confidence intervals. Intervals lying to the right of zero indicate positive Δ*r* and those to the left indicate negative Δ*r*. Seventeen of the 20 CIs include zero; the single positive exclusion (F6c, NUDT15 stability) is isolated and is addressed as a multiple-comparisons issue in Section “Statistical significance testing”. c) Forest plot of Δ*r* = *r*_F7_−r_F2_ for the 4 F7 multitask protein–task combinations (Table 7). Two of the 4 CIs exclude zero, both in the direction of reduced performance, and the mean absolute effect across the 4 comparisons is 0.015. d) Test Pearson *r* with fold standard-deviation error bars for F2, F8-MLP (structure-agnostic ablation), F8-GCN, and F9-GAT across all 5 datasets (Table 8). F8-MLP closely tracks F2 (4 of 5 datasets within CI), but both F8-GCN and F9-GAT substantially underperform F2 in all 5 datasets (arrows indicate the magnitude of the GCN decrement below F2). The F8-MLP control localizes the GCN/GAT performance loss to message passing rather than to pipeline differences.

**Table 6.** Paired significance testing of F6 structural feature variants against the F2 sequence-only baseline.

Dataset	Variant	Δ*r*	95% CI	Wilcoxon *P*	Interval excludes zero?
CYP2C9 activity	F6a	−0.019	[−0.027, −0.005]	0.125	Yes
CYP2C9 activity	F6b	−0.009	[−0.032, +0.012]	0.813	No
CYP2C9 activity	F6c	−0.016	[−0.032, −0.001]	0.313	Yes
CYP2C9 activity	F6d	−0.007	[−0.038, +0.021]	0.813	No
CYP2C9 abundance	F6a	−0.002	[−0.016, +0.013]	1.000	No
CYP2C9 abundance	F6b	+0.017	[−0.007, +0.039]	0.313	No
CYP2C9 abundance	F6c	−0.018	[−0.040, +0.010]	0.313	No
CYP2C9 abundance	F6d	−0.009	[−0.034, +0.014]	0.438	No
CYP2C19 abundance	F6a	+0.007	[−0.002, +0.016]	0.313	No
CYP2C19 abundance	F6b	−0.002	[−0.030, +0.026]	0.813	No
CYP2C19 abundance	F6c	+0.006	[−0.003, +0.015]	0.313	No
CYP2C19 abundance	F6d	−0.007	[−0.026, +0.014]	1.000	No
NUDT15 stability	F6a	−0.007	[−0.025, +0.012]	0.625	No
NUDT15 stability	F6b	−0.009	[−0.041, +0.024]	0.813	No
NUDT15 stability	F6c	+0.025	[+0.005, +0.049]	0.125	Yes
NUDT15 stability	F6d	+0.019	[−0.007, +0.051]	0.313	No
NUDT15 sensitivity	F6a	−0.009	[−0.024, +0.008]	0.313	No
NUDT15 sensitivity	F6b	−0.010	[−0.038, +0.011]	0.813	No
NUDT15 sensitivity	F6c	+0.006	[−0.020, +0.027]	0.813	No
NUDT15 sensitivity	F6d	−0.008	[−0.029, +0.012]	0.625	No

For each dataset–variant combination, the mean paired difference in test Pearson *r* across 5 folds (Δ*r* = *r*_F6_−*r*_F2_) is shown with 95% bootstrap confidence intervals (10,000 resamples) and Wilcoxon signed-rank *P*-values. Significance is assessed by CI exclusion alone, since the joint criterion is unattainable at 5 folds; 3 intervals exclude zero, 2 negative and one positive.

Three of the 20 F6 variant–dataset combinations have intervals excluding zero, 2 in the negative direction and 1 positive (F6c, NUDT15 stability), the latter consistent with the false-positive rate expected across 20 comparisons. Positive Δ*r* values were observed in 6 combinations and negative Δ*r* in 14, with a mean |Δ*r*| across all combinations of 0.011, well within the typical fold-to-fold variance of F2 itself (±0.024–0.062).

The consistent near-zero mean differences (|Δ*r*| = 0.011 across all 20 combinations, comparable to the ±0.024–0.062 fold-to-fold variance of F2 itself) indicate that naive structural feature augmentation does not provide a measurable advantage for pharmacogene variant effect prediction at this scale. The specific featurization that most directly approximates explicit 3D information—F6b, neighbor ESM-2 pooling—did not systematically outperform the simpler featurizations, suggesting that the information content of the mean neighbor embedding is largely redundant with the ESM-2 embedding at the variant position itself. The F6d full-concatenation variant (2,634 dimensions) likewise did not outperform the more targeted variants, a pattern that parallels the feature ablation results in Section “Feature ablation of the supervised baseline”, although as noted there the contributions of feature content and model capacity cannot be separated in either comparison.

These results are consistent with prior evidence that transformer protein language models implicitly encode substantial structural information through co-evolutionary patterns in their training data (Rives et al. 2021; Lin et al. 2023). For pharmacogene variant effect prediction, this implicit structural encoding appears to saturate the information available from AlphaFold-predicted monomeric contact graphs when the downstream supervised model is a shallow MLP. Whether explicit graph neural network modeling of the same contact structure can recover additional signal beyond simple feature concatenation is addressed in Section “Contact-graph message passing over frozen embeddings degrades prediction”.

### Multitask learning yields no detectable improvement and degrades 2 of 4 tasks

To test whether jointly modeling paired phenotypic assays improves variant effect prediction over single-task training, a multitask architecture (F7) was evaluated on the 2 protein-level dataset pairs for which both assays were measured on the same variants, namely CYP2C9 (activity and abundance; *n* = 4,421 paired variants) and NUDT15 (stability and thiopurine sensitivity; *n* = 2,844 paired variants). F7 extends the F2 baseline by sharing a two-layer feature backbone (1,320 → 512 → 256) across tasks and attaching 2 task-specific heads (256 → 64 → 1), trained end-to-end with a weighted-sum loss L = 0.5·MSE_A + 0.5·MSE_B on targets *z*-score-normalized within each training fold (Section “Multitask learning architecture”). All other training hyperparameters (position-based 5-fold cross-validation, Adam optimizer, learning rate 1 × 10^−3^, dropout 0.2, early stopping with patience 12) were held identical to F2 to enable a direct paired comparison.

The 2 task pairs differ markedly in phenotypic concordance. In CYP2C9, activity and abundance scores correlate strongly (raw Pearson *r* = 0.748 across paired variants), consistent with the VAMP-seq abundance assay capturing folding-dependent steady-state protein levels that largely determine catalytic output. In NUDT15, stability (VAMP-seq) and thiopurine sensitivity correlate weakly (*r* = 0.384), reflecting that a subset of variants perturb substrate binding or catalysis without destabilizing the protein. A priori, multitask learning would be expected to benefit most from correlated tasks (CYP2C9) and to risk negative transfer in decoupled tasks (NUDT15).

Neither expectation was met in the direction of benefit. None of the 4 protein-task combinations improved under joint training, and 2 of the 4 showed a measurable cost. In CYP2C9, joint training reduced performance on activity prediction (Δ*r* = −0.011, 95% CI [−0.014, −0.007]) and left abundance unchanged (Δ*r* = + 0.001, 95% CI [−0.016, +0.021]), so the strong phenotypic correlation between the 2 assays conferred no advantage. In NUDT15, the effect was asymmetric: stability prediction degraded substantially (Δr = −0.043, 95% CI [−0.069, −0.022]) while thiopurine sensitivity was unaffected (Δ*r* = +0.006, 95% CI [−0.008, +0.017]). The 2 intervals excluding 0 therefore correspond to the largest degradation occurring in the decoupled pair, which is consistent with the negative-transfer expectation, and to a smaller but still detectable degradation in the correlated pair, which is not. Wilcoxon signed-rank tests returned *P* = 0.0625 for both degraded tasks, the minimum value attainable with 5 folds, so interval-based significance is the operative criterion here (Section “Statistical significance testing”). The negative transfers were consistent rather than fold-driven, since the per-fold difference was negative in all 5 folds for both affected tasks; this consistency is what yields confidence intervals narrower than the fold-to-fold standard deviation of either model taken alone (0.021 to 0.061 across the 4 F2 references, Table 7), because the paired bootstrap resamples differences and removes the fold-level variation common to both models. The mean absolute effect across all 4 comparisons was 0.015. Full results are summarized in Table 7 and illustrated in Fig. 3c.

**Table 7.** Paired significance testing of F7 multitask learning against the F2 single-task baseline.

Protein	Task	F2 r	F7 r	Δr	95% CI	Wilcoxon *P*	Interval excludes zero?
CYP2C9	Activity	0.700 ± 0.021	0.689 ± 0.021	−0.011	[−0.014, −0.007]	0.0625	Yes
CYP2C9	Abundance	0.688 ± 0.039	0.689 ± 0.029	+0.001	[−0.016, +0.021]	1.0000	No
NUDT15	Stability	0.602 ± 0.061	0.559 ± 0.075	−0.043	[−0.069, −0.022]	0.0625	Yes
NUDT15	Sensitivity	0.688 ± 0.022	0.694 ± 0.034	+0.006	[−0.008, +0.017]	0.6250	No

For each protein–task combination, the mean paired difference in test Pearson *r* across 5 position-based CV folds (Δ*r* = r_F7_−*r*_F2_) is shown with 95% bootstrap confidence intervals (10,000 resamples) and Wilcoxon signed-rank *P*-values. Values were regenerated under the corrected fold-local normalization procedure described in Section “Multitask learning architecture”. With 5 folds the minimum attainable Wilcoxon *P*-value is 0.0625, so interval-based significance is the primary criterion. No combination shows improvement under joint training, and 2 intervals exclude zero, both in the direction of reduced performance.

These results extend the earlier finding that explicit structural feature augmentation (F6, Section “Structural feature augmentation yields no detectable improvement over the sequence-only baseline”) also failed to improve over F2 (no systematic improvement across 20 comparisons). In both cases the additional modeling capacity, whether structural context from AlphaFold contact graphs or shared representations across paired assays, did not translate into gains on held-out positions, and joint training additionally imposed a measurable cost on 2 of the 4 tasks. Taken together, these outcomes are consistent with the interpretation that, within the model classes evaluated here and at the dataset sizes available, the additional inductive biases introduced by structural augmentation and multitask learning were largely redundant with the structural and functional information already encoded implicitly in the ESM-2 embedding, and that forcing a shared representation across assays measuring different biochemical properties can interfere with the task-specific signal rather than merely failing to enhance it.

These findings should nonetheless be interpreted alongside 2 methodological caveats. The multitask architecture used here relies on a fixed equal-weight joint loss (*α* = 0.5) without adaptive task weighting, whereas approaches such as uncertainty-weighted multitask learning (Kendall et al. 2018) or GradNorm (Chen et al. 2018) could in principle mitigate the task-imbalance effects that the asymmetric transfer in NUDT15 suggests, where stability degraded substantially while thiopurine sensitivity did not. Because the benefit of multitask learning typically scales with the number of related tasks, the signal available for transfer is in any case modest with only 2 paired assays per protein.

### Contact-graph message passing over frozen embeddings degrades prediction

Although structural feature augmentation (F6, Section “Structural feature augmentation yields no detectable improvement over the sequence-only baseline”) failed to improve over F2, those augmentations relied on hand-constructed summary statistics of the local structural neighborhood, leaving open the possibility that explicit graph neural network modeling, which learns neighborhood aggregation as part of end-to-end training, could extract additional signal from the AlphaFold contact graph. To address this possibility, 2 GNN architectures (F8-GCN and F9-GAT; Section “Graph neural network architectures”) were evaluated alongside a matched structure-agnostic ablation (F8-MLP) in which the same node-encoder architecture was used but the graph edges were removed. All 3 models share the identical node featurization (1,281-dimensional ESM-2 plus pLDDT concatenation) and prediction head (hidden + 40 → 128 → 64 → 1, where the 40 additional inputs are the wild-type and mutant one-hot encodings), differing only in whether and how residue–residue messages are propagated.

Hyperparameters were selected by grid search on the NUDT15 stability dataset (8 configurations per model class; Section “Graph neural network architectures”), yielding a hidden dimension of 256 with 2 layers and a learning rate of 1 × 10^−3^ for F8-MLP (*r* = 0.580 ± 0.068 on the grid dataset), a hidden dimension of 128 with 3 layers and a learning rate of 1 × 10^−3^ for F8-GCN (*r* = 0.431 ± 0.053), and a hidden dimension of 256 with 2 layers and a learning rate of 1 × 10^−3^ for F9-GAT (*r* = 0.455 ± 0.099). These settings were fixed across all 5 datasets to avoid optimistic bias from per-dataset tuning. Because the grid search used mean test-fold performance rather than a held-out validation split (Section “Graph neural network architectures”), the absolute values reported for all 3 model classes should be read as optimistic; the comparison between them is unaffected, since all 3 were selected under the same procedure.

The 5-fold position-based cross-validation results for F8-MLP, F8-GCN, and F9-GAT, together with paired bootstrap significance tests against both the F2 sequence baseline (Sections “Model architecture and Feature ablation of the supervised baseline”) and F8-MLP (the structure-agnostic ablation), are summarized in Table 8 and illustrated in Fig. 3d.

**Table 8.** Graph neural network evaluation against sequence baselines.

Dataset	F2 r	F8-MLP r	F8-GCN r	F9-GAT r	Δ*r* (MLP − F2) [95% CI]	Δ*r* (GCN − F2) [95% CI]	Δ*r* (GAT − F2) [95% CI]	Δ*r* (GCN − MLP) [95% CI]	Δ*r* (GAT − MLP) [95% CI]
CYP2C9 activity	0.682 ± 0.024	0.680 ± 0.039	0.455 ± 0.065	0.526 ± 0.061	−0.002 [−0.021, +0.018]	**−0.227 [−0.275, −0.179]**	**−0.156 [−0.190, −0.112]**	**−0.225 [−0.261, −0.192]**	**−0.154 [−0.174, −0.130]**
CYP2C9 abundance	0.679 ± 0.026	0.698 ± 0.038	0.561 ± 0.027	0.642 ± 0.058	+0.019 [−0.002, +0.039]	**−0.118 [−0.153, −0.089]**	**−0.037 [−0.074, −0.002]**	**−0.137 [−0.171, −0.099]**	**−0.056 [−0.078, −0.034]**
CYP2C19 abundance	0.713 ± 0.022	0.745 ± 0.026	0.511 ± 0.049	0.682 ± 0.019	**+0.032 [+0.013, +0.053]**	**−0.202 [−0.254, −0.148]**	**−0.031 [−0.058, −0.001]**	**−0.234 [−0.284, −0.194]**	**−0.063 [−0.086, −0.040]**
NUDT15 stability	0.557 ± 0.062	0.580 ± 0.068	0.431 ± 0.053	0.455 ± 0.099	+0.023 [−0.013, +0.058]	**−0.126 [−0.179, −0.072]**	**−0.102 [−0.175, −0.029]**	**−0.148 [−0.206, −0.098]**	**−0.125 [−0.177, −0.074]**
NUDT15 sensitivity	0.666 ± 0.024	0.679 ± 0.020	0.447 ± 0.075	0.517 ± 0.023	+0.013 [−0.004, +0.039]	**−0.219 [−0.293, −0.154]**	**−0.148 [−0.183, −0.113]**	**−0.232 [−0.294, −0.164]**	**−0.161 [−0.184, −0.129]**

Five-fold position-based cross-validation test Pearson *r* (mean ± SD) for F8-MLP (structure-agnostic ablation), F8-GCN, and F9-GAT, compared pairwise against the F2 sequence-only baseline, retrained within this pipeline on identical folds, and against F8-MLP. For each comparison, the mean paired difference Δ*r* and its 95% bootstrap confidence interval (10,000 resamples) are reported. Wilcoxon signed-rank *P*-values are shown but constrained by the minimum achievable value at *n* = 5 folds (*p*_min_ = 0.0625). Statistical significance is assessed by whether the bootstrap CI excludes zero.

Bold indicates comparisons whose 95% bootstrap CI excludes zero. By the CI-based significance criterion, F8-MLP differs significantly from F2 in only 1 of 5 datasets (positive direction), whereas F8-GCN and F9-GAT both underperform F2 in all 5 datasets, and both underperform F8-MLP in all 5 datasets as well. Across the 10 GNN-vs-F2 comparisons (F8-GCN and F9-GAT combined), none represents a statistically significant improvement, whereas all 10 represent statistically significant underperformance by the bootstrap CI criterion. Wilcoxon signed-rank *P*-values reach their minimum possible value (0.0625) for most of these bootstrap-significant comparisons, reflecting a statistical floor imposed by *n* = 5 paired folds rather than an absence of effect.

The structure-agnostic F8-MLP ablation closely tracked the F2 baseline across all 5 datasets, with mean paired differences Δ*r* = *r*_F8-MLP_−r_F2_ ranging from −0.002 (CYP2C9 activity) to +0.032 (CYP2C19 abundance) and a 95% bootstrap CI including zero in 4 of 5 datasets. The single dataset on which F8-MLP exceeded F2 with a CI excluding zero was CYP2C19 abundance (Δ*r* = +0.032, 95% CI [+0.013, +0.053]), a modest improvement plausibly attributable to the larger hidden width (256 vs 256/64 in F2) permitted by the simpler feature dimension. This behavior anchors the comparison: because the GNN node-encoder pipeline reproduces F2 performance when edges are removed, any observed difference between F8-GCN or F9-GAT and F8-MLP can be attributed to explicit message passing rather than to upstream pipeline or featurization differences.

Against this backdrop, both graph neural network variants substantially underperformed F2. F8-GCN produced a negative Δ*r* on all 5 datasets with every bootstrap interval below zero, ranging from −0.118 to −0.227 Pearson *r*, and F9-GAT followed the same pattern with milder decrements of −0.031 to −0.156 (Table 8). The same pattern holds against F8-MLP, the structure-agnostic control sharing identical featurization, with every interval excluding zero for both operators (Table 8), so the loss is attributable to the graph convolution itself rather than to the modified pipeline. Within this implementation, message passing over the static contact graph degrades prediction relative to a matched no-structure control, and does so under both operators tested, symmetric-normalized (GCN) and attention-weighted (GAT). The GAT attention mechanism recovers part of the gap relative to GCN (mean Δ*r*_GAT − Δ*r*_GCN = +0.08 averaged across datasets), consistent with attention weighting suppressing some fraction of uninformative neighbors, but without eliminating the performance penalty of message passing.

Taken together with the F6 structural augmentation (Section “Structural feature augmentation yields no detectable improvement over the sequence-only baseline”) and F7 multitask (Section “Multitask learning yields no detectable improvement and degrades 2 of 4 tasks”) findings, these GNN results form a consistent picture in which none of the 3 architectural extensions evaluated here provides a significant improvement over the F2 sequence-only baseline, and 2 of them (F8-GCN and F9-GAT) actively harm performance. The structure-agnostic F8-MLP ablation clarifies that the lack of improvement is not due to node-feature degradation or pipeline artifact but is localized to the message-passing step as implemented here, namely spectral or attention-weighted convolution over a static 8 Å Cα contact graph with frozen ESM-2 node features. A plausible mechanism is signal dilution: the ESM-2 embedding at the variant position encodes local sequence and implicit structural context with high specificity, whereas graph convolution averages this high-specificity signal across topological neighbors whose variant-level phenotypes may differ substantially. For pharmacogene DMS prediction at current dataset scales, on the order of 3,000 to 8,000 labeled variants per protein, this spatial averaging appears to remove more signal than it adds. These results bear on the specific combination of a static monomeric contact graph, comparatively simple GCN and GAT operators, and frozen node features, and not on structure-aware modeling in general; architectures that encode geometry rather than topology, that condition on ligands, or that fine-tune the underlying representation were not evaluated here (Section “Graph neural network architectures”).

### The baseline identifies damaging variants but not the dimension along which they are damaging

Pearson correlation measures agreement with a continuous assay readout, whereas pharmacogenomic interpretation asks 2 narrower questions: whether a variant is severely functionally impaired, and through which biochemical dimension the impairment acts. The F2 baseline was evaluated against both, using the held-out predictions from the benchmark described above (Section “Classification and function-specific detection metrics”).

On the first question, the baseline performs well. Across the 5 datasets it separates severely impaired variants from the remainder with AUROC between 0.835 and 0.917, and AUPRC between 0.580 and 0.784 against prevalence floors of 0.150 to 0.354, so the ranking carries substantial information beyond the class balance in every case (Table 9). Precision among the 50 variants ranked most damaging is 0.88 or higher on 4 of the 5 datasets, which is the regime relevant to prioritizing candidates for experimental follow-up. Calibration slopes lie between 0.88 and 1.06 with small positive intercepts, indicating that predictions are close to the identity relation with a mild overstatement of severity at the damaging extreme. The one clear exception is NUDT15 thiopurine sensitivity, where AUROC is high (0.911) but precision among the top 50 falls to 0.64, meaning that the variants the model ranks as most damaging are frequently not those the assay records as most sensitive. This is the assay that depends on substrate handling rather than folding, and the same task was the only one on which the supervised baseline failed to exceed the ESM1v zero-shot ensemble (Section “Comparison with zero-shot and pretrained baselines”). It is also the dataset on which Spearman falls furthest below Pearson (*ρ* = 0.580 against *r* = 0.668; Supplementary Table 6), so 3 independent measures of rank quality converge on the same conclusion for this assay.

**Table 9.** Detection of severe functional loss by the F2 baseline.

Dataset	*N*	*N* severe (%)	AUROC	AUPRC	AUPRC floor	*P*@50	*P*@100	*P*@200	Calib. slope	Calib. intercept
CYP2C9 activity	6,142	2,177 (35.4%)	0.868 ± 0.017	0.784 ± 0.041	0.354	0.94	0.92	0.90	0.932	0.089
CYP2C9 abundance	6,370	956 (15.0%)	0.917 ± 0.031	0.691 ± 0.077	0.150	0.88	0.90	0.85	0.882	0.148
CYP2C19 abundance	7,830	1,281 (16.4%)	0.901 ± 0.013	0.638 ± 0.052	0.164	0.90	0.84	0.81	0.892	0.132
NUDT15 stability	2,922	832 (28.5%)	0.835 ± 0.034	0.706 ± 0.028	0.285	0.94	0.93	0.87	0.990	0.116
NUDT15 sensitivity	2,934	454 (15.5%)	0.911 ± 0.016	0.580 ± 0.068	0.155	0.64	0.66	0.61	1.059	0.027

Variants with a measured score below 0.3 are the positive class. AUROC and AUPRC are computed within each held-out test fold and averaged across the 5 folds (mean ± SD); the AUPRC floor is the positive-class prevalence. Precision@k is computed on the pooled held-out predictions. Calibration slope and intercept are from the regression of measured on predicted score.

On the second question, the baseline fails, and the failure is the more informative result. For CYP2C9, the 2 single-task models predict activity and abundance at *r* = 0.667 and 0.683 respectively, yet the difference between their predictions tracks the measured activity–abundance difference at only *r* = 0.284 (95% CI [0.250, 0.318]). Separating the 1,236 discordant variants from the concordant remainder reaches an AUROC of 0.600 (95% CI [0.582, 0.619]), and for NUDT15 the corresponding value is 0.511 (95% CI [0.490, 0.532]) with an AUPRC of 0.482 against a prevalence floor of 0.480, that is, no measurable gain over ranking at random. The directional categories are recovered somewhat better, with an AUROC of 0.694 for the CYP2C9 stable-but-dead category and 0.668 for the NUDT15 paradoxical-resistant category, but precision remains low: AUPRC of 0.093 against a floor of 0.044 for the former and 0.120 against 0.078 for the latter (Table 10). Two models trained separately on 2 assays of the same protein therefore reproduce each assay individually while largely failing to reproduce the quantity that distinguishes them.

**Table 10.** Recovery of function-specific variant effects from paired assays.

	CYP2C9 (activity vs abundance)	NUDT15 (sensitivity vs stability)
Paired variants	4,421	2,844
*r*, predicted vs measured assay difference	0.284 [0.250, 0.318]	0.433 [0.402, 0.464]
Discordant variants (|measured diff| > 0.3)	1,236 (28.0%)	1,364 (48.0%)
Discordant AUROC	0.600 [0.582, 0.619]	0.511 [0.490, 0.532]
Discordant AUPRC (floor)	0.378 (0.280)	0.482 (0.480)
Directional category	stable-but-dead, 195 (4.4%)	paradoxical-resistant, 222 (7.8%)
Category AUROC	0.694 [0.659, 0.728]	0.668 [0.636, 0.700]
Category AUPRC (floor)	0.093 (0.044)	0.120 (0.078)

For each variant measured in both assays of a protein, the difference between the 2 independently trained single-task F2 predictions is compared with the measured assay difference. Confidence intervals are percentile bootstrap intervals over variants (2,000 resamples). AUPRC floors are the prevalence of the corresponding class.

For reference, the same models predict the individual assays at Pearson *r* = 0.667 (CYP2C9 activity), 0.683 (CYP2C9 abundance), 0.668 (NUDT15 sensitivity), and 0.554 (NUDT15 stability) in the rerun described in Section “Classification and function-specific detection metrics”, which reproduces Table 3 to within 0.017.

This result connects the 2 halves of the study. The paired assays establish that function-specific effects are numerically dominant (Section “Functional heterogeneity in CYP2C9 and NUDT15”), and the AlphaMissense stratification establishes that a general-purpose pathogenicity score does not resolve them (Section “A general-purpose pathogenicity predictor does not separate biochemically distinct variant categories”). The present analysis shows that the failure is not confined to predictors trained on a different objective: a supervised model trained directly on the assay of interest, which predicts that assay competently, still does not recover the dimension along which a variant acts once the 2 assays are compared. The information required to make that distinction appears not to be present in the wild-type sequence representation used here, which is consistent with the absence of gains from multitask joint training on exactly these paired assays (Section “Multitask learning yields no detectable improvement and degrades 2 of 4 tasks”).

## Discussion

Pharmacogene missense variants act along multiple biochemical dimensions, and the paired deep mutational scanning data analyzed here show that this multidimensionality is quantitatively dominant rather than marginal. The benchmark reported here, spanning sequence-based, structure-augmented, multitask, and graph-based deep learning approaches under a single position-based cross-validation protocol, was designed to establish whether that dimensionality is recoverable with current methods. The resulting picture converges on 3 mutually reinforcing observations that together constrain what is currently tractable for pharmacogene variant effect prediction. Each observation is examined in turn below, beginning with the empirical magnitude of phenotypic decoupling between paired biochemical assays, continuing with the divergence between this biochemistry and the scores assigned by a pan-protein pathogenicity predictor, and concluding with the finding that none of the 3 architectural extensions evaluated here recovered additional signal beyond the ESM-2 sequence baseline at current dataset scales, with 2 of them reducing accuracy relative to it.

The magnitude of functional heterogeneity recovered from paired CYP2C9 and NUDT15 assays is large enough to be biologically meaningful rather than reducible to assay noise or scoring normalization. For CYP2C9, 1,236 of 4,421 paired variants (28%) exhibit absolute activity–abundance differences exceeding 0.3 normalized units, and the joint Pearson correlation of 0.748 reproduces the value of 0.76 independently obtained by Boyle et al. (2024), thereby providing cross-laboratory confirmation of the data ingestion and parsing pipeline. The decoupling concentrates at residues with established biochemical roles such as Ser365, Glu300, Ala297, Val113, and Cys435, all of which fall within the substrate-binding channel that governs the metabolism of warfarin, flurbiprofen, celecoxib, and phenytoin (Theken et al. 2020; Boyle et al. 2024). This pattern aligns with the broader observation that cytochrome P450 catalytic specificity is shaped not only by active-site residues but also by an interconnected network of access channels lined with phenylalanine gating residues that discriminate substrates before they reach the buried heme (Fishelovitch et al. 2009; Urban et al. 2018). Across the human proteome, recent work using ESM-derived disentangling models estimates that approximately 45% of pathogenic missense variants act through stability loss while the remaining 33% perturb function through other mechanisms (Cagiada et al. 2025), placing the 28% function-specific fraction observed here within the upper end of the expected biochemical range for an enzyme whose catalytic competence depends on substrate channel integrity rather than fold stability alone. The corresponding NUDT15 statistic is even more pronounced, since 1,364 of 2,844 paired variants (48%) decouple stability from thiopurine sensitivity, and a substantial subset shows the paradoxical pattern of destabilization coupled to preserved or enhanced thiopurine metabolism. The structural basis for this decoupling has been mechanistically elucidated for the most clinically prevalent NUDT15 variant, R139C, which retains intrinsic catalytic activity in vitro yet undergoes accelerated proteasomal degradation due to loss of supportive intramolecular bonds (Carter et al. 2015; Rehling et al. 2021). Given that NUDT15 loss-of-function is the principal pharmacogenetic determinant of thiopurine-induced myelosuppression in patients receiving azathioprine or 6-mercaptopurine for inflammatory bowel disease, acute lymphoblastic leukemia, or transplant immunosuppression (Maillard et al. 2024), the clinically actionable distinction is therefore not whether a variant is damaging but through which mechanism the damage is realized, and that distinction lies beyond the reach of any single-scalar predictor.

The same decoupling that emerges from the paired-assay analysis also explains why a predictor trained on clinical pathogenicity scores these biochemically defined categories in the way it does. Stratification of CYP2C9 joint-assay variants reveals 38 of 195 stable-but-dead variants (19%) receiving AlphaMissense scores in the “likely benign” range despite complete catalytic inactivation in the Click-seq assay, with these variants concentrating at residues Asn202, Asp188, Ile169, Glu203, Thr392, Lys241, Leu170, Leu197, Leu29, and Cys175. The opposite pattern dominates the NUDT15 paradoxical-resistant category, where 222 variants destabilize the protein yet retain wild-type-like thiopurine sensitivity scores, and AlphaMissense assigns them a median pathogenicity score of 0.67 with 63% labeled “likely pathogenic”. If such scores were used in place of functional annotation in a dosing decision, they would point toward thiopurine reduction for variants whose assayed phenotype suggests, if anything, the opposite, though whether this would occur in practice depends on clinical evidence that the present data cannot supply. This pattern is not unique to AlphaMissense. Independent benchmarking on 530 pharmacogenetic variants from CPIC and high-quality functional databases reports an AlphaMissense sensitivity of only 33% despite specificity of 94% (Zhou et al. 2019; Curtis 2024), and analogous overcalling has been documented for CFTR variants, where AlphaMissense shows high false positive rates and limited utility for distinguishing theratype-relevant biochemical mechanisms (Mcdonald et al. 2024). A separate evaluation against 17,696 MAVE-derived variants across DDX3X, MSH2, PTEN, KCNQ4, and BRCA1 likewise concludes that AlphaMissense and related predictors overcall variants as pathogenic despite minimal functional impact (Ljungdahl et al. 2023). The explanation is consistent across these reports and is one of objective rather than of quality, since AlphaMissense was trained to predict general clinical pathogenicity, an objective that aligns well with protein-level folding disruption but poorly with substrate-specific catalytic behavior or with the altered-function phenotypes that dominate the CPIC star-allele framework, where alleles are categorized along a graded scale spanning no, decreased, normal, increased, and uncertain function (Theken et al. 2020). The CPIC NSAID guideline explicitly notes that the catalytic activity of CYP2C9 decreased function and no function alleles is substrate-dependent and that allele function assignment requires careful evaluation of individual drugs (Theken et al. 2020), a regulatory acknowledgment that pan-protein pathogenicity scoring cannot substitute for substrate-resolved functional annotation. Recent architectures such as PreMode, which targets mode-of-action prediction (loss-of-function vs gain-of-function) using SE(3)-equivariant graph neural networks, address part of this gap but still operate at the binary pathogenicity level rather than at the substrate-specific resolution required for pharmacogene dosing (Zhong et al. 2025).

The methodologically most striking finding from this benchmark is the convergent failure of 3 architectural extensions to surpass the F2 sequence-only baseline, with 2 of them actively degrading it. Structural feature augmentation drawing on AlphaFold contact graphs yielded a mean |Δ*r*| of 0.011, comparable to the fold-to-fold standard deviation of F2 itself, with the single positive interval isolated to one dataset and treated as a multiple-comparisons artifact. Multitask joint learning across paired phenotypic assays (F7) likewise failed to improve over single-task F2 in any of the 4 protein–task combinations, including the high-correlation CYP2C9 activity–abundance pair (raw *r* = 0.748) where positive transfer would have been expected most strongly, and in 2 of those combinations joint training significantly reduced accuracy, with confidence intervals excluding zero for CYP2C9 activity (Δ*r* = −0.011) and NUDT15 stability (Δ*r* = −0.043). The graph neural network extensions went further, underperforming F2 in all 10 comparisons, and the structure-agnostic F8-MLP ablation isolated that loss to the graph convolution itself rather than to any pipeline or featurization artifact. The most plausible mechanistic explanation invokes signal dilution. The ESM-2 per-residue embedding at the variant position encodes local sequence and implicit structural context with high specificity, while neighborhood aggregation across 8 Å topological neighbors averages this high-specificity signal across residues whose variant-level phenotypes may differ substantially. This phenomenon parallels the well-characterized over-smoothing pathology of deep graph neural networks, in which repeated message passing drives node representations toward indistinguishability and degrades downstream task performance (Li et al. 2018). Analogous findings have accumulated in protein function and DMS fitness prediction. Villegas-Morcillo et al. found that contact-graph GCNs fall below sequence-only baselines once amino acid embeddings come from pretrained protein language models (Villegas-Morcillo et al. 2021), and Wang et al. (2022) reached the same conclusion when comparing GCN- and GAT-based architectures under the LM-GVP framework. Boca and Mathis extended this observation to DMS fitness prediction, showing that SE(3)-equivariant geometric graph neural networks such as GVP and EQGAT yield only modest improvements over Tranception sequence baselines despite their substantially richer geometric inductive bias (Boca and Mathis 2023). Protein language models pretrained at evolutionary scale appear to already encode substantial tertiary structural context through co-evolutionary patterns (Rives et al. 2021), and explicit message passing over a static 8 Å Cα contact graph therefore becomes redundant or actively counterproductive at the dataset scales examined here, which span 2,922 to 7,830 labeled variants per protein. This pattern is not confined to protein variant effect prediction. Sasse et al. found that sequence-to-expression models predicting gene expression from DNA perform well across genomic regions yet frequently fail to recover the direction of effect of cis-regulatory variants across individuals, which is structurally the same observation as the one reported here: an aggregate metric is satisfied while the quantity carrying the interpretive meaning is not (Sasse et al. 2023). The recurring lesson in both settings is that the evaluation must be posed at the resolution the application requires.

One reading of the feature ablation results deserves to be stated as an interpretation rather than as a conclusion. Across variants F1 to F5, hidden-layer width was tied to input dimensionality, so the higher-dimensional representations were consumed by wider networks with substantially more parameters, and neither factor was varied independently. One plausible reading is that a shallow fully connected network cannot exploit spatial structure presented through simple input concatenation, since both the window and the full-concatenation variants underperformed the minimal representation. An equally plausible reading is that the additional parameters introduced by the wider first layer, roughly 10 times as many under F3 as under F2, exceeded what several thousand labeled variants can constrain, so that the loss reflects estimation error rather than an absence of usable signal in the features. Distinguishing the 2 would require holding hidden-layer width fixed while varying only the input representation, which the design used here does not do.

Bringing these 3 threads together produces a useful null hypothesis for future methodological work in this domain. Any proposed extension, whether architectural, featural, or supervisory, should be benchmarked against an ESM-2-plus-MLP sequence baseline under position-based cross-validation with paired bootstrap significance testing before its incremental value can be claimed. The reference point established here, with F2 reaching Pearson *r* between 0.54 and 0.72 across 5 datasets and matching or marginally exceeding both ESM1v zero-shot ensembles and supervised AlphaMissense predictions, provides a concrete baseline against which methodological refinement can be calibrated within this problem domain.

The clinical implication is sharper still, and it is not confined to predictors trained on a mismatched objective. A supervised model trained directly on each assay identifies severely impaired variants well (AUROC 0.835 to 0.917) yet recovers the difference between 2 assays of the same protein at *r* = 0.284 for CYP2C9 and 0.433 for NUDT15, and separates the discordant class at an AUROC of 0.600 and 0.511 respectively, the latter indistinguishable from chance (Section “The baseline identifies damaging variants but not the dimension along which they are damaging”). Using pan-protein pathogenicity scores in place of pharmacogene-specific functional annotation would leave at least 2 biochemically defined variant categories unresolved, and the 38 CYP2C9 stable-but-dead variants and 222 NUDT15 paradoxical-resistant variants identified in this study constitute named, specific candidates for experimental follow-up and for inclusion in curated pharmacogene catalogs such as PharmVar, subject to the patient-level evidence that such curation requires.

Placing this output on the path to clinical curation requires being explicit about which resource it can serve. PharmVar catalogs pharmacogene variation and defines star alleles as haplotypes but does not assign function; the designations on its gene pages are CPIC's, made by gene-specific Pharmacogene Curation Expert Panels on a graded scale spanning no, decreased, normal, increased, uncertain, and unknown function (Gaedigk et al. 2018; Tibben et al. 2025). That framework maintains a separate designation of allele biochemical function which is not used to translate genotypes into predicted phenotypes and which can diverge from the clinical assignment, CYP2C9*3 being no function clinically while retaining low but measurable activity biochemically (Tibben et al. 2025). The measurements reported here speak to the biochemical tier, not the clinical one. Three obstacles separate them from a curation-ready submission. Star alleles are haplotypes while DMS resolves single substitutions, so a score informs a haplotype's core missense variant without characterizing the haplotype. Calibration is absent: the ClinGen framework for functional evidence requires validation against variants of established effect and conversion to an odds of pathogenicity (Brnich et al. 2019), whereas these assays are normalized to wild-type and nonsense, which gives an interpretable scale but not a calibrated one. Locating the alleles that already carry CPIC assignments within the DMS distribution, and asking whether the no-function, decreased-function, and normal-function designations occupy separable ranges, is the most direct next step. Third, CPIC's CYP2C9 assignment is explicitly substrate-dependent (Theken et al. 2020), and a single-substrate Click-seq score cannot be mapped onto a substrate-agnostic label.

NUDT15 makes the argument concrete. Its PharmVar annotation already reports in vitro activity toward 2 thiopurine substrates alongside thermostability as a separate indicator of function, so the two-dimensional structure documented here is one the field already maintains, at present for a handful of designated alleles rather than for 2,844 measured variants. The 2025 CPIC thiopurine guideline further notes that no clinically available pharmacological phenotype exists for NUDT15 genotype, placing more weight on in vitro evidence for this gene than for TPMT (Relling et al. 2019; Maillard et al. 2026). The defensible use of saturation data is therefore prospective triage rather than direct assignment: they cover far more coding sequence than has received star-allele designation, so a newly identified haplotype will usually have a core substitution with both an abundance and a substrate-handling score already measured, and the paired readout indicates not merely whether function is reduced but whether the reduction is a folding defect or an alteration in substrate handling. That distinction determines whether an assignment should be expected to hold across substrates. The 38 CYP2C9 stable-but-dead and 222 NUDT15 paradoxical-resistant variants are candidates for exactly this triage, being those at which a general score and an assay-specific measurement disagree.

A question this benchmark raises without answering is whether a model trained jointly across paralogous pharmacogenes would perform better than the per-gene models evaluated here, and whether principles learned on one cytochrome P450 transfer to another. The data assembled for this study permit a limited version of that test which was not attempted: CYP2C9 and CYP2C19 are close paralogs sharing the cytochrome P450 fold, and both were assayed for abundance by VAMP-seq, so a model trained on one and evaluated on the other would measure directly how much of the determinants of folded abundance is shared. Two difficulties would have to be handled. Scores from different studies are normalized independently to wild-type and nonsense controls, so absolute values are not interchangeable and a joint model would have to be trained on ranks or on per-dataset standardized targets. More fundamentally, a joint model must learn what transfers and what does not, and the results reported here suggest the 2 are quite different: fold-level determinants of abundance are conserved across the family, whereas the substrate channel residues at which CYP2C9 activity decouples from abundance are precisely the residues whose substrate preferences differ between paralogs. Transfer would therefore be expected to help on the abundance axis and to hurt on the activity axis, which is the same asymmetry seen in the multitask results within a single protein.

Whether to supply that structure as a prior or to learn it from data is the second half of the question. For cytochrome P450 enzymes the priors are unusually concrete and annotatable across the whole family: the heme-coordinating cysteine, the I-helix acid–alcohol pair, the substrate access channels, and the N-terminal membrane anchor are defined for every member. A model conditioned on these annotations would not have to rediscover them from a few thousand labeled variants. The structural feature augmentation evaluated here (F6c) is a weak and unannotated version of this idea, encoding neighborhood composition and geometry rather than functional role, and it did not improve on the sequence baseline, so whether explicit functional annotation would fare better remains open. The gene classes most amenable to this approach are those with many paralogs sharing a fold and a cofactor, of which the cytochrome P450 family is the clearest example within pharmacogenomics, followed by the UDP-glucuronosyltransferases and the solute carrier transporters. The binding constraint in every case is the same: at least 2 family members assayed on a common platform, which at present exists only for CYP2C9 and CYP2C19 abundance.

Closing the gap between what these predictors return and what pharmacogene interpretation requires may run through the acquisition of richer supervision signals rather than through further refinement of the model classes evaluated here, and 3 data modalities appear particularly promising in this regard, although model classes outside the scope of this benchmark may also contribute. Multisubstrate DMS, in which the same variant library is assayed against multiple physiologically relevant substrates, would expose substrate-specific variant effects directly and supply the supervision signal required for substrate-conditional prediction models that explicitly encode small-molecule chemistry. The feasibility of this approach has now been demonstrated at scale. D-amino acid oxidase has been profiled across 5 substrates with approximately 6,500 unique variants using enzyme proximity sequencing (Vanella et al. 2024), and phospho-PCA has enabled simultaneous measurement of stability, catalytic activity, and substrate specificity for approximately 15,000 variants across 3 closely related kinases (Fyn, Lck, Src) using Bayesian disentangling of the joint phenotypic signal (Yu et al. 2025). Adapting these approaches to CYP2C9, which metabolizes warfarin, flurbiprofen, celecoxib, and phenytoin through partially overlapping but biochemically distinct mechanisms, would directly address the substrate-specific decoupling reported here and would provide training data for the next generation of chemistry-aware predictors that combine protein language model embeddings with chemical language model representations of small-molecule substrates (Daly et al. 2017). A second promising modality involves double-mutant deep mutational scans, given that clinically actionable pharmacogene alleles such as CYP2D6 *4, CYP2C9 *2/*3, and NUDT15 *3 are defined at the haplotype level and frequently involve co-occurring variants whose functional consequences are nonadditive. Systematic active-site epistasis scans of CTX-M-14 β-lactamase have revealed that 3.2% of double mutant pairs exhibit positive epistasis often mediated through substrate interactions rather than direct residue contacts (Judge et al. 2024), and analogous combinatorial scans of myoglobin, fluorescent proteins, and protein–protein interfaces have generated training data for machine learning models that predict double-mutant fitness from single-mutant measurements (Küng et al. 2026). Extending this paradigm to clinically relevant pharmacogenes would enable haplotype-level functional prediction of the *2/*3/*4 allele structures that dominate CPIC dosing guidelines. Higher-throughput clinical pharmacogenomic phenotyping constitutes the third modality, given that integrating DMS-derived functional scores with patient-derived metabolic ratios would permit supervised learning directly from clinical endpoints rather than from biochemical surrogates. The infrastructure for this integration is now in place. Pharmacogenetic analyses of 487,409 UK Biobank participants have established that 99.5% of individuals carry at least one variant predicted to alter response to a commonly prescribed drug, with non-European populations carrying a greater frequency of functionally deleterious variants poorly captured by existing star-allele definitions (Mcinnes et al. 2021), and EHR-based frameworks combining UK Biobank and All of Us data have begun to systematically link genotype to drug-response phenotype across cardiometabolic medications (Sadler et al. 2025).

A series of methodological boundaries should be borne in mind when interpreting the findings reported above and when designing follow-up work, and these are organized below into 8 themes spanning the scope of the model classes evaluated, dataset scope, model selection, statistical sensitivity, structural input fidelity, cross-validation semantics, assay-specific ground truth, and the single-variant framing of the benchmark. The scope of the model classes evaluated is the first and most immediate boundary. This study compares a supervised ESM-2-plus-MLP baseline against the ESM1v zero-shot ensemble and AlphaMissense, and extends that baseline with AlphaFold-derived structural features, multitask learning, and comparatively simple GCN and GAT operators, but it is not an exhaustive survey of contemporary variant effect prediction. On the representation side, the supervised models use wild-type ESM-2 embeddings together with wild-type and mutant amino acid encodings, and do not evaluate mutant-sequence embeddings, delta embeddings, or likelihood-ratio features derived from more recent protein language models, nor lightweight fine-tuning strategies such as adapter or low-rank updates to the language model itself. On the modeling side, unsupervised generative approaches such as EVE, non-neural supervised methods including gradient-boosted trees, ligand-aware architectures that condition on small-molecule substrate representations, and rotation-equivariant geometric networks such as GVP-GNN, EquiformerV2, and GearNet were likewise outside the scope of this benchmark. The negative results reported here therefore apply to the specific model classes evaluated under the specific datasets available, and should not be read as evidence that variant effect prediction for pharmacogenes has reached a general performance limit. Dataset scope constitutes a second and equally consequential boundary. This benchmark covers 3 pharmacogenes for which paired biochemical DMS data are publicly available through MaveDB, namely CYP2C9, CYP2C19, and NUDT15, but does not include CYP2D6, which metabolizes approximately one-quarter of all clinically prescribed drugs and is arguably the most pharmacologically important member of the cytochrome P450 family. Brown et al. (2022) explicitly noted that DMS has not yet been employed for CYP2D6 phenotype prediction, and the catalog of pharmacogenes with paired biochemical DMS assays remains small. As a consequence, the performance range reported here (Pearson *r* = 0.54 to 0.72 for the F2 sequence baseline) is empirically anchored only to the 5 assays examined and its generalization to CYP2D6 and to other clinically actionable pharmacogenes (TPMT, UGT1A1, SLCO1B1, DPYD) remains an open question. The convergent-negative pattern observed for architectural extensions (F6, F7, F8-GCN, F9-GAT) is likewise conditioned on the datasets analyzed; a DMS dataset an order of magnitude larger, or one in which substrate-specific effects dominate at a greater fraction of positions, might recover signal from contact graphs or multitask joint learning that proved unrecoverable at present scales.

Model selection constitutes a further boundary, and one that applies to 2 separate steps. The graph neural network hyperparameters were chosen by grid search on the NUDT15 stability dataset using mean test-fold performance rather than a held-out validation split, and the feature configuration carried forward as the supervised baseline was likewise the best performer on test folds across the 5 datasets. Neither departure was repeated under a corrected protocol. The direction of the resulting bias is nonetheless conservative with respect to the negative conclusions drawn here, since in both cases the configuration was chosen to maximize apparent performance on the data against which it is subsequently evaluated: the graph models were tuned to look as favorable as possible and still underperformed the matched structure-agnostic control in all 10 comparisons, so a correctly validated search would leave that conclusion unchanged or strengthen it. What the bias does compromise is the absolute values, which should be read as optimistic for every model class whose configuration was selected in this way. The concern was partly anticipated for the graph models, whose selected configuration was deliberately fixed across all 5 datasets without per-dataset tuning to avoid compounding the selection bias, but that mitigation does not remove the departure itself.

Statistical sensitivity also bounds the strength of the negative findings. With 5 cross-validation folds, the paired Wilcoxon signed-rank test has a minimum achievable *P*-value of 2/2^5^ = 0.0625, below the conventional *α* = 0.05 threshold regardless of effect magnitude (Methods Section “Statistical significance testing”), and primary reliance therefore falls on paired bootstrap 95% confidence intervals as the significance criterion, which are not subject to this minimum-*P* floor. Bootstrap power to detect small but real effects is itself limited by the number of folds, since the smallest mean differences plausibly flaggable as significant correspond to roughly ±0.015 to ±0.020 Pearson *r* units given typical fold-to-fold standard deviations of 0.02 to 0.06. Genuine architectural improvements smaller than this could be masked by fold-level variance, and the claim that no significant improvement arises from F6/F7/F8/F9 extensions is therefore conditional on effect sizes exceeding this detection floor. Increasing the number of folds, for instance through leave-one-position-out cross-validation, which would yield 163 to 486 paired comparisons per dataset, would substantially improve sensitivity at proportional computational cost, and represents a natural extension of the present benchmark.

Structural input fidelity introduces another boundary. The contact graphs employed here are derived from AlphaFold-predicted monomeric structures (AFDB v6), and although AlphaFold provides high-confidence backbone predictions for the 3 proteins examined (mean pLDDT 92.7 to 94.2; Table 2), monomeric models do not capture biologically relevant features that depend on membrane anchoring (cytochrome P450 enzymes are endoplasmic reticulum membrane proteins), heme coordination geometry (central to CYP catalysis), allosteric conformational ensembles, or protein–protein complexes with cytochrome P450 reductase. The contact graphs analyzed therefore encode the average static fold rather than the dynamic, ligand- and substrate-coupled conformations that determine catalytic specificity in vivo. This limitation may itself contribute to the observed underperformance of the graph neural networks evaluated here (F8-GCN, F9-GAT), since an 8 Å Cα–Cα contact graph of a single static fold is likely a poor approximation of the information content actually useful for predicting substrate-specific variant effects. Experimental or dynamically sampled structures such as molecular dynamics ensembles, cryo-EM maps with bound substrates, and NMR-derived conformational ensembles would offer a more biochemically faithful substrate for structure-based modeling, although obtaining such data at pharmacogene scale remains substantially more costly than relying on AlphaFold predictions.

Cross-validation semantics constitute a further boundary. The position-based protocol eliminates within-position label leakage but cannot fully eliminate structural leakage in graph-based models, because the AlphaFold contact graph and the ESM-2 embedding are computed from the complete wild-type sequence and structure, such that features of held-out positions remain visible to the model during training even though the functional labels at those positions are not. This is consistent with the intended deployment scenario of such models, namely prospective prediction for a new variant at a residue position whose wild-type structure is already known, but it means that the reported test-set correlations measure generalization under the assumption of complete structural and sequence context rather than true held-out-residue extrapolation. For the sequence-only F2 baseline this is a standard feature of ESM-2-based prediction; for the graph-based models (F8-GCN, F9-GAT), the F8-MLP structure-agnostic ablation isolates the incremental effect of message passing, but any leakage via the graph structure itself remains implicit and is difficult to fully remove without retraining the protein language model with fold-specific residue masking, an expensive intervention not attempted here.

Assay-specific ground truth introduces an additional caveat. Each DMS assay is treated here as ground truth for the biochemical quantity of interest (catalytic activity, folded abundance, thiopurine sensitivity), but each carries assay-specific biases that shape the variant-effect distributions observed. The VAMP-seq abundance assays used for CYP2C9, CYP2C19, and NUDT15 depend on folding-mediated degradation and may under-detect noncanonical stable misfolds; the Click-seq activity assay reports hydroxylation of a single model substrate and cannot capture activity toward the full panel of clinical substrates that CYP2C9 metabolizes (warfarin, flurbiprofen, celecoxib, phenytoin) in vivo. The 1,236 function-specific CYP2C9 variants and 1,364 substrate-specific NUDT15 variants reported here are therefore defined relative to the specific biochemical assays of Amorosi et al. (2021) and Suiter et al. (2020), and their clinical actionability requires independent validation against orthogonal biochemical assays or patient-derived pharmacokinetic data. The stratified AlphaMissense analysis (Section 3.6) inherits this limitation, since the 38 likely benign CYP2C9 stable-but-dead variants and the 222 likely pathogenic NUDT15 paradoxical-resistant variants are specific, named candidates for follow-up, but their ultimate clinical classification depends on experimental or clinical evidence beyond the DMS ground truth assumed here. A further dimension is absent from these assays altogether. VAMP-seq reports steady-state folded abundance through degradation-coupled fluorescence and does not report where in the cell the protein accumulates, so a variant that folds, escapes degradation, and yet fails to reach its compartment would register as abundance-preserved while being nonfunctional in vivo. This is not a hypothetical concern for the proteins studied here, since cytochrome P450 enzymes are targeted to the endoplasmic reticulum through an N-terminal signal-anchor sequence and their catalytic cycle requires colocalization with cytochrome P450 reductase in the same membrane. Multiplexed assays with imaging-based subcellular readouts would resolve this third axis, and the expectation from the present results is that it would extend rather than overturn the argument made here: the 2 dimensions resolved by the paired assays are already sufficient to defeat a single scalar score, and a third would only widen the gap between what a variant effect predictor returns and what pharmacogene interpretation requires.

The single-variant framing of the benchmark constitutes the final and arguably most fundamental boundary. The structure of currently available DMS datasets supports only single-substitution analysis, but clinically actionable pharmacogene alleles (CYP2D6 *4, CYP2C9 *2/*3, NUDT15 *3) are defined at the haplotype level and frequently involve multiple co-occurring variants whose functional consequences can be nonadditive (Scott et al. 2021). No single-variant predictor, whether sequence-based, structure-based, or multitask, can capture such epistatic interactions by construction, and the F2 baseline, along with AlphaMissense, ESM1v, and every other predictor benchmarked here, is therefore intrinsically limited to providing a first-pass functional annotation that must be integrated with haplotype-level reasoning downstream. Extending DMS to systematic double-mutant scans, as discussed above, is the natural experimental path toward supervised haplotype-level prediction, but the requisite datasets do not yet exist at the scale needed for robust supervised modeling across the clinical pharmacogene panel.

These boundaries shape the scope of the claims made above without undermining the central contribution of this work, namely the demonstration that pharmacogene missense variants frequently act along separable biochemical dimensions that current predictors do not resolve, together with a reproducible benchmark against which future methods can be evaluated under the position-based cross-validation and paired bootstrap protocol adopted here.

## Conclusion

Across 26,198 missense variants in CYP2C9, CYP2C19, and NUDT15, separation between protein-level and function-level variant effects proves to be the rule rather than the exception. Paired biochemical assays reveal that 28% of CYP2C9 variants and 48% of NUDT15 variants decouple protein stability from catalytic or substrate-specific activity, with function-specific effects concentrating at residues of known biochemical consequence in the CYP2C9 substrate-binding channel. This heterogeneity reflects the distinct molecular mechanisms through which a pharmacogene variant can reach clinical significance rather than an artifact of assay normalization, and it defines the interpretive problem that the benchmark reported here was designed to test.

The consequences of ignoring this dimensionality are concrete and named. AlphaMissense, taken as a representative general-purpose pathogenicity predictor, scored 38 of 195 CYP2C9 stable-but-dead variants as benign despite complete catalytic inactivation in the assay, and assigned pathogenic classifications to 140 of 222 NUDT15 paradoxical-resistant variants that retain wild-type-like thiopurine sensitivity scores. These divergences follow from a training objective directed at clinical pathogenicity rather than at assay-specific function, and they mark the points at which a single generic score and a pharmacogene-specific functional measurement part company. The specific variants identified here therefore constitute a testable candidate set for experimental follow-up and for incorporation into curated resources such as PharmVar and the CPIC allele function tables.

The benchmark was designed to test whether current computational approaches can recover this dimensionality, and no such recovery was detectable under the evaluated design. A simple ESM-2 embedding paired with a two-layer MLP achieves Pearson *r* between 0.54 and 0.72 across the 5 datasets, matching or marginally exceeding both the ESM1v zero-shot ensemble and supervised AlphaMissense predictions. None of the 3 architectural extensions improved upon this baseline, and 2 imposed a measurable cost: graph convolution degraded performance across all 10 comparisons, and joint training reduced accuracy on 2 of 4 tasks. A structure-agnostic ablation localizes the graph result to the message-passing step, consistent with dilution of a sequence representation that already encodes substantial implicit structural context.

Within the model classes evaluated here, methodological refinement at the current dataset scale did not close the gap that the stratified analyses expose. What these data point toward instead is richer supervision through multisubstrate DMS that exposes substrate-specific decoupling as a trainable signal, through systematic double-mutant scans that make clinically defined haplotypes tractable at the supervised level, and through integration with patient-derived pharmacokinetic endpoints from population biobanks. Until such datasets become available, the baseline reported here provides a concrete reference point against which any proposed extension should be calibrated under paired bootstrap significance testing before its incremental value can be claimed. For the model classes examined here, the limiting factor appears to lie less in the architecture than in the biochemical resolution of the training signal itself, a proposition that further benchmarking across a wider range of approaches would help to test.

## Supplementary Material

jkag248_Supplementary_Data

## Data Availability

The dataset and code are publicly available at https://github.com/tissueandcells/pharmacogene-dms-benchmark. The repository contains the full analysis pipeline, including data ingestion scripts, ESM-2 embedding extraction, feature construction (F1–F6), multitask (F7) and graph neural network (F8/F9) training code, position-based 5-fold cross-validation protocol, paired bootstrap significance testing, and figure-generation notebooks. All deep mutational scanning datasets analyzed in this study are publicly accessible through the MaveDB API (Rubin et al. 2025; https://www.mavedb.org) under the accession identifiers listed in Table 1. AlphaFold structures are available from the AlphaFold Protein Structure Database (Varadi et al. 2024; https://alphafold.ebi.ac.uk). Supplemental material available at G3 online.
